# Genetic effects influencing risk for major depressive disorder in China and Europe

**DOI:** 10.1038/tp.2016.292

**Published:** 2017-03-28

**Authors:** T B Bigdeli, S Ripke, R E Peterson, M Trzaskowski, S-A Bacanu, A Abdellaoui, T F M Andlauer, A T F Beekman, K Berger, D H R Blackwood, D I Boomsma, G Breen, H N Buttenschøn, E M Byrne, S Cichon, T-K Clarke, B Couvy-Duchesne, N Craddock, E J C de Geus, F Degenhardt, E C Dunn, A C Edwards, A H Fanous, A J Forstner, J Frank, M Gill, S D Gordon, H J Grabe, S P Hamilton, O Hardiman, C Hayward, A C Heath, A K Henders, S Herms, I B Hickie, P Hoffmann, G Homuth, J-J Hottenga, M Ising, R Jansen, S Kloiber, J A Knowles, M Lang, Q S Li, S Lucae, D J MacIntyre, P A F Madden, N G Martin, P J McGrath, P McGuffin, A M McIntosh, S E Medland, D Mehta, C M Middeldorp, Y Milaneschi, G W Montgomery, O Mors, B Müller-Myhsok, M Nauck, D R Nyholt, M M Nöthen, M J Owen, B W J H Penninx, M L Pergadia, R H Perlis, W J Peyrot, D J Porteous, J B Potash, J P Rice, M Rietschel, B P Riley, M Rivera, R Schoevers, T G Schulze, J Shi, S I Shyn, J H Smit, J W Smoller, F Streit, J Strohmaier, A Teumer, J Treutlein, S Van der Auwera, G van Grootheest, A M van Hemert, H Völzke, B T Webb, M M Weissman, J Wellmann, G Willemsen, S H Witt, D F Levinson, C M Lewis, N R Wray, J Flint, P F Sullivan, K S Kendler

**Affiliations:** 1 Department of Psychiatry, Virginia Institute for Psychiatric and Behavioral Genetics, Virginia Commonwealth University School of Medicine, Richmond, VA, USA; 2 Department of Psychiatry, Charite Universitatsmedizin Berlin Campus Benjamin Franklin, Berlin, Germany; 3 Medical and Population Genetics, Broad Institute, Cambridge, MA, USA; 4 Analytic and Translational Genetics Unit, Massachusetts General Hospital, Boston, MA, USA; 5 Institute for Molecular Bioscience, The University of Queensland, Brisbane, QLD, Australia; 6 Queensland Brain Institute, The University of Queensland, Brisbane, QLD, Australia; 7 Department of Biological Psychology, Vrije Universiteit Amsterdam, Amsterdam, The Netherlands; 8 Department of Translational Research in Psychiatry, Max Planck Institute of Psychiatry, Munich, Germany; 9 Munich Cluster for Systems Neurology (SyNergy), Munich, Germany; 10 Department of Psychiatry, VU University Medical Center and GGZ inGeest, Amsterdam, The Netherlands; 11 Institute of Epidemiology and Social Medicine, University of Muenster, Münster, Germany; 12 Division of Psychiatry, University of Edinburgh, Edinburgh, UK; 13 King's College London, NIHR BRC for Mental Health, London, UK; 14 King's College London, MRC Social Genetic and Developmental Psychiatry Centre, London, UK; 15 Department of Clinical Medicine, Translational Neuropsychiatry Unit, Aarhus University, Aarhus, Denmark; 16 Department of Biomedicine, University of Basel, Basel, Switzerland; 17 Division of Medical Genetics, University of Basel, Basel, Switzerland; 18 Institute of Neuroscience and Medicine (INM-1), Research Center Juelich, Jülich, Germany; 19 Institute of Human Genetics, University of Bonn, Bonn, Germany; 20 Department of Genetics and Computational Biology, QIMR Berghofer Medical Research Institute, Brisbane, QLD, Australia; 21 Centre for Advanced Imaging, University of Queensland, Brisbane, QLD, Australia; 22 Department of Psychological Medicine, Cardiff University, Cardiff, UK; 23 EMGO+ Institute, VU University Medical Center, Amsterdam, The Netherlands; 24 Department of Genomics, Life and Brain Center, University of Bonn, Bonn, Germany; 25 Stanley Center for Psychiatric Research, Broad Institute, Cambridge, MA, USA; 26 Department of Psychiatry, Massachusetts General Hospital, Boston, MA, USA; 27 Psychiatric and Neurodevelopmental Genetics Unit, Massachusetts General Hospital, Boston, MA, USA; 28 Department of Psychiatry and Behavioral Sciences, State University of New York Downstate Medical Center, Brooklyn, NY, USA; 29 Department of Genetic Epidemiology in Psychiatry, Central Institute of Mental Health, Medical Faculty Mannheim, Heidelberg University, Heidelberg, Germany; 30 Department of Psychiatry, Trinity College Dublin, Dublin, Ireland; 31 Department of Psychiatry and Psychotherapy, University Medicine Greifswald, Greifswald, Germany; 32 Department of Psychiatry, Kaiser-Permanente Northern California, San Fransisco, CA, USA; 33 Institute of Neuroscience, Trinity College Dublin, Dublin, Ireland; 34 Medical Research Council Human Genetics Unit, Institute of Genetics and Molecular Medicine, University of Edinburgh, Edinburgh, UK; 35 Department of Psychiatry, Washington University in Saint Louis School of Medicine, St Louis, MO, USA; 36 Division of Medical Genetics, Department of Biomedicine, University of Basel, Basel, Switzerland; 37 Brain and Mind Research Institute, University of Sydney, Sydney, NSW, Australia; 38 Human Genomics Research Group, Department of Biomedicine, University of Basel, Basel, Switzerland; 39 Department of Functional Genomics, Interfaculty Institute for Genetics and Functional Genomics, University Medicine and Ernst Moritz Arndt University Greifswald, Greifswald, Germany; 40 Max Planck Institute of Psychiatry, Munich, Germany; 41 Department of Psychiatry and The Behavioral Sciences, University of Southern California, Los Angeles, CA, USA; 42 Neuroscience Therapeutic Area, Janssen Research and Development, LLC, Titusville, NJ, USA; 43 Department of Psychiatry, Washington University in Saint Louis School of Medicine, St Louis, MO, USA; 44 School of Psychology, University of Queensland, Brisbane, QLD, Australia; 45 Department of Psychiatry, New York State Psychiatric Institute, Columbia University College of Physicians and Surgeons, New York, NY, USA; 46 Centre for Cognitive Ageing and Cognitive Epidemiology, University of Edinburgh, Edinburgh, UK; 47 Institute for Molecular Biology, University of Queensland, Brisbane, QLD, Australia; 48 Psychosis Research Unit, Aarhus University Hospital, Risskov, Denmark; 49 Department of Molecular and Clinical Pharmacology, University of Liverpool, Liverpool, UK; 50 Institute of Clinical Chemistry and Laboratory Medicine, University Medicine Greifswald, Greifswald, Germany; 51 Institute of Health and Biomedical Innovation, Queensland University of Technology, Brisbane, QLD, Australia; 52 MRC Centre for Neuropsychiatric Genetics and Genomics, Cardiff University School of Medicine, Cardiff, UK; 53 Charles E. Schmidt College of Medicine, Florida Atlantic University, Boca Raton, FL, USA; 54 Department of Psychiatry, Harvard Medical School, Boston, MA, USA; 55 Department of Psychiatry, Massachusetts General Hospital, Boston, MA, USA; 56 Medical Genetics Section, CGEM, IGMM, University of Edinburgh, Edinburgh, UK; 57 Department of Psychiatry, University of Iowa, Iowa, IA, USA; 58 Department of Psychiatry, Washington University in Saint Louis, St Louis, MO, USA; 59 Department of Human and Molecular Genetics, Virginia Commonwealth University School of Medicine, Richmond, VA, USA; 60 Department of Biochemistry and Molecular Biology II, Institute of Neurosciences, Center for Biomedical Research, University of Granada, Granada, Spain; 61 King's College London, MRC Social Genetic and Developmental Psychiatry Centre, London, UK; 62 Department of Psychiatry, University of Groningen, University of Medical Center Groningen, Groningen, The Netherlands; 63 Institute of Psychiatric Phenomics and Genomics, Medical Center of the University of Munich, Campus Innenstadt, Munich, Germany; 64 Department of Psychiatry and Psychotherapy, University Medical Center Göttingen, Göttingen, The Netherlands; 65 Department of Psychiatry and Behavioral Sciences, Johns Hopkins University, Baltimore, MD, USA; 66 Human Genetics Branch, NIMH Division of Intramural Research Programs, Bethesda, MD, USA; 67 Division of Cancer Epidemiology and Genetics, National Cancer Institute, Bethesda, MD, USA; 68 Division of Psychiatry, Group Health, Seattle, WA, USA; 69 Institute for Community Medicine, University Medicine Greifswald, Greifswald, Germany; 70 Department of Psychiatry, Leiden University Medical Center, Leiden, The Netherlands; 71 Division of Epidemiology, New York State Psychiatric Institute, New York, NY, USA; 72 Department of Psychiatry, Columbia University College of Physicians and Surgeons, New York, NY, USA; 73 Psychiatry and Behavioral Sciences, Stanford University, Stanford, CA, USA; 74 King's College London, Department of Medical and Molecular Genetics, London, UK; 75 Merton College, University of Oxford, Oxford, UK; 76 Wellcome Trust Centre for Human Genetics, University of Oxford, Oxford, UK; 77 Medical Epidemiology and Biostatistics, Karolinska Institutet, Solna, Sweden; 78 Department of Genetics, University of North Carolina at Chapel Hill, Chapel Hill, NC, USA; 79 Department of Psychiatry, University of North Carolina at Chapel Hill, Chapel Hill, NC, USA

## Abstract

Major depressive disorder (MDD) is a common, complex psychiatric disorder and a leading cause of disability worldwide. Despite twin studies indicating its modest heritability (~30–40%), extensive heterogeneity and a complex genetic architecture have complicated efforts to detect associated genetic risk variants. We combined single-nucleotide polymorphism (SNP) summary statistics from the CONVERGE and PGC studies of MDD, representing 10 502 Chinese (5282 cases and 5220 controls) and 18 663 European (9447 cases and 9215 controls) subjects. We determined the fraction of SNPs displaying consistent directions of effect, assessed the significance of polygenic risk scores and estimated the genetic correlation of MDD across ancestries. Subsequent trans-ancestry meta-analyses combined SNP-level evidence of association. Sign tests and polygenic score profiling weakly support an overlap of SNP effects between East Asian and European populations. We estimated the trans-ancestry genetic correlation of lifetime MDD as 0.33; female-only and recurrent MDD yielded estimates of 0.40 and 0.41, respectively. Common variants downstream of *GPHN* achieved genome-wide significance by Bayesian trans-ancestry meta-analysis (rs9323497; log_10_ Bayes Factor=8.08) but failed to replicate in an independent European sample (*P*=0.911). Gene-set enrichment analyses indicate enrichment of genes involved in neuronal development and axonal trafficking. We successfully demonstrate a partially shared polygenic basis of MDD in East Asian and European populations. Taken together, these findings support a complex etiology for MDD and possible population differences in predisposing genetic factors, with important implications for future genetic studies.

## Introduction

Major depressive disorder (MDD) is the most common psychiatric illness and a leading cause of disability worldwide.^1, 2^ MDD is modestly heritable (30–40%), may be genetically complex and likely heterogeneous, complicating efforts to identify replicable risk loci.^3, 4^ The successful detection and interpretation of genetic associations require both increased sample sizes^5^ and empirically driven efforts to reduce phenotypic heterogeneity.^6^

Underpinning the success of genome-wide association studies (GWAS) of numerous traits has been the emergence of large research consortia.^7^ In addition to facilitating larger sample sizes, many consortia are increasingly ancestrally diverse, enabling identification of novel associations^8, 9, 10^ and independent replication of reported findings,^11, 12^ as well as improving fine mapping of implicated loci.^13, 14^ Consistent associations at replicated loci have been reported for psychiatric disorders^15^ and non-psychiatric traits,^8, 11, 16, 17, 18^ and shared liabilities are often borne out by genome-wide polygenic analyses.^19, 20, 21^

Whether genetic factors predisposing to MDD are shared across ancestries is not well established, and two replicated genome-wide significant associations for MDD in China had markedly lower allele frequencies in Europeans and thus did not replicate.^22, 23, 24^ Allelic heterogeneity and population-specific genetic effects have been reported for several complex traits;^18, 25, 26^ however, the extent of differences across ancestries remains relatively unexplored.

We sought to clarify the extent to which liability to MDD is shared between European and East Asian populations via collaboration between the Psychiatric Genomics Consortium (PGC)^22^ and CONVERGE^6^ studies of MDD. We asked whether observed directions of allelic effects are consistent across populations, assessed the significance of cross-ancestry polygenic scores and estimated the trans-ancestry genetic correlation of MDD. We attempted to disentangle population differences from those arising from ascertainment or phenotypic definition through analyses of recurrent MDD and in female subjects. These meta-analyses represent the largest trans-ancestry genetic study of MDD to date.

## Materials and methods

### Ascertainment and genotyping

Sample ascertainment, SNP genotyping and quality-control procedures for PGC and CONVERGE have been described previously.^6, 22^ Individual sites and sample sizes are presented in Table 1.

*CONVERGE* (**C**hina, **O**xford a**n**d **V**irginia Commonwealth University **E**xperimental **R**esearch on **G**enetic **E**pidemiology): Briefly, all subjects were Han Chinese women and had two or more episodes of MDD meeting DSM-IV criteria. After applying quality controls modeled after the PGC study, 10 502 samples (5282 cases and 5220 controls) and 6 242 619 SNPs were retained for analysis.

*PGC MDD:* Samples included here comprise Stage 1 of the PGC MDD study.^22^ Briefly, all subjects were of European ancestry, all cases were assessed using validated methods and met DSM-IV criteria for lifetime MDD, and the majority of controls were screened to exclude lifetime MDD. Available data on number of depressive episodes were used to identify recurrent cases (two or more episodes). Nine studies from the US, Europe and Australia were genotyped using SNP arrays. Imputation was performed with IMPUTE2 (ref. 27) using the 1000 Genomes Project data (v3; GRCh37/hg19),^28^ resulting in a total of 13 381 627 autosomal and X chromosome SNPs.

### Polygenic risk score profiling and binomial sign tests

Each data set was filtered on the basis of statistical imputation information (INFO) greater than 0.8 and minor allele frequency greater than 0.01 in both CONVERGE and PGC overall; linkage disequilibrium (LD)-based 'clumping' was used to obtain an approximately independent set of SNPs (*r*^2^<0.1) while preferentially retaining the most significant SNP within 500-kb windows. We computed weighted polygenic scores (that is, log odds ratio of the associated allele), based on varying *P*-value thresholds in the 'training set' results (that is, CONVERGE or PGC); *P*-value thresholds ranged between 10^−^^5^ and 0.5. We evaluated the significance of case–control differences using logistic regression and covarying ancestry-based principal components and a study indicator variable. The predictive value of these scores is reported in terms of Nagelkerke's pseudo-*R*^2^ (fmsb package in R).^29^

Using the same sets of SNPs and the same *P*-value thresholds, we applied a binomial sign test to determine whether the number of SNPs demonstrating consistent directions of allelic effects between CONVERGE and PGC was greater than expected by chance (that is, a one-sided test of whether this fraction is greater than 0.5).

### Trans-ancestry genetic correlation

The recently developed *popcorn* software^30^ allows for estimation of the trans-ancestry genetic effect correlation (*ρ*_g_) using GWAS summary statistics. Cross-ancestry reference scores, representing SNP-wise estimates of the similarity of LD (with neighboring SNPs) between populations, were calculated for East Asian (*N*=286) and European (*N*=379) subjects from the 1000 Genomes Project (v3).^28^ For computational efficiency and consistency with previously reported estimates of genetic correlation, these calculations were based on ~1.2 M common SNPs present in HapMap3 (ref. 31) following study-wise exclusion of SNPs with INFO<0.9 or minor allele frequency<0.01%.

We attempted to address possible heterogeneity by examining estimates of genetic correlation within- and across-ancestries, and for varying phenotypic definitions. Briefly, we divided the PGC and CONVERGE studies into approximate halves, performing association analysis in each subsample as described above, and subsequently estimating the genetic correlations between these nonoverlapping halves. Within the PGC, we randomly selected 5 of 10 studies (*S*_EUR1_), with the remaining five studies taken as a comparison sample (*S*_EUR2_). We selected *N*=30 of a possible 126 paired comparisons for which the sample sizes of each subset were equivalent (~1:1). We followed an analogous procedure in CONVERGE, selecting 12 of 24 sequencing batches (*S*_ASN1_), with the remaining 12 batches taken as a comparison sample (*S*_ASN2_). Within-ancestry comparisons were between nonoverlapping subsets (for example, *S*_EUR1_ versus *S*_EUR2_) and utilized reference scores based on a single population, calculated as described above. Cross-ancestry estimates were based on comparisons of the full set of CONVERGE results to each of *N*=60 subsets from the within-PGC analysis. We compared cross-ancestry estimates for lifetime MDD, recurrent MDD and females-only by paired Student's *t*-tests.

### SNP-based meta-analyses

Within each study, we tested for association between SNPs and affection status by logistic regression with PLINK,^32^ using allelic dosages and including ancestry principal components as covariates (plus a site indicator in PGC analyses). Backward-stepwise regression was used to select principal components demonstrating association (*P*<0.159) with each diagnosis. We excluded SNPs with minor allele frequency<0.01 or INFO<0.5 in either CONVERGE or PGC (overall), or missing in greater than equal to five of nine PGC samples. We analyzed the X chromosome as previously described.^22^

We performed Bayesian meta-analyses of PGC and CONVERGE studies using MANTRA.^33^ By leveraging population differences in local LD structure, MANTRA has greater power to detect genetic effects demonstrating allelic heterogeneity than traditional approaches assuming random effects. When effects are consistent across studies, MANTRA is effectively a Bayesian implementation of fixed-effects meta-analysis. Interstudy genetic distances were calculated from the mean allele frequency differences. We adopted a threshold of log_10_ Bayes factor (log_10_BF) >7 for declaring genome-wide significance.

### Gene-set enrichment analyses

We applied DEPICT^34^ to identify significantly enriched gene sets and pathways in specific tissues and cell types. Briefly, genes in the vicinity of associated SNPs are tested for enrichment for 'reconstituted' gene sets, comprising curated sets expanded to include co-regulated loci. Tissue and cell-type enrichment analysis is conducted by testing whether genes were highly expressed in any of 209 MeSH annotations based on microarray data for the Affymetrix U133 Plus 2.0 Array platform (Santa Clara, CA, USA).^35^

Because DEPICT adjusts for potential sources of confounding and multiple testing using precomputed GWAS of randomly distributed phenotypes, we elected to use as input *P*-values from inverse variance weighted (that is, fixed effects) meta-analysis of PGC and CONVERGE. Recalling that MANTRA is effectively a Bayesian implementation of fixed-effects meta-analysis when allele frequencies are similar between populations, we considered this to be an appropriate strategy, if not somewhat conservative.

### Replication analyses

A total of 4504 cases and 7007 controls from 10 independent, European-ancestry cohorts were available for replication (Table 1). These studies represent recent additions to the PGC that were not included in the previously published analysis.^22^ A brief description of each study site is given in the Supplementary Material. At the time of writing, neither comparable East Asian GWAS data sets nor subject-level data on the number of depressive episodes were readily available. For analyses of recurrent illness, we included those replication studies that specifically ascertained recurrent cases.

For each phenotype definition, we identified independent (pairwise *r*^2^<0.1 within 500-kb windows based on European 1000 Genomes Project samples), significant autosomal SNPs (log_10_BF>5) from the trans-ancestry meta-analyses (10, 7 and 7 for MDD, female-only and recurrent MDD, respectively). We tested these SNPs for association using logistic regression and including ancestry principal components as covariates. We performed inverse-variance weighted meta-analyses of the replication samples using METAL. We also performed binomial sign tests comparing the directions of allelic effects across discovery and replication stages.

## Results

### Polygenic risk score profiling and binomial sign tests

**Figure 1:**
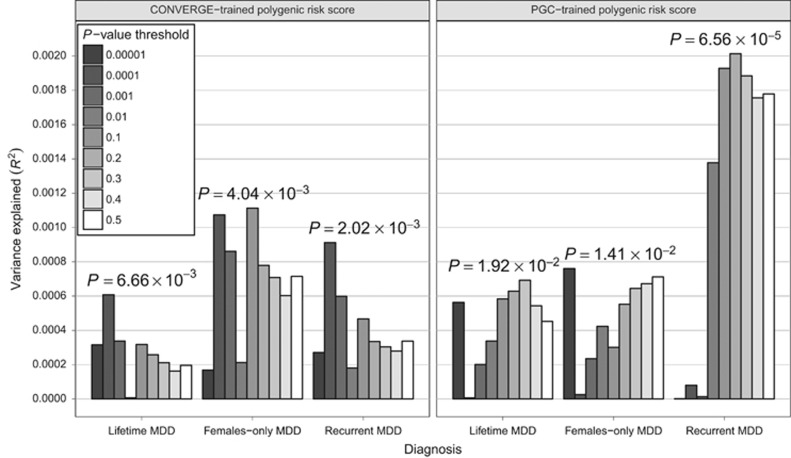
Trans-ancestry association of polygenic risk scores with major depressive disorder**.** For scores based on results from PGC or CONVERGE, the variance in risk explained in the other study is shown on the *y* axis in terms of Nagelkerke's pseudo-*R*^2^; scores based on various *P*-value inclusion thresholds are displayed as shaded bars. CONVERGE, China, Oxford and Virginia Commonwealth University Experimental Research on Genetic Epidemiology; MDD, major depressive disorder; PGC, Psychiatric Genomics Consortium.

We employed polygenic risk score profiling to determine whether findings from CONVERGE or the PGC are, in aggregate, significantly associated with the MDD status in the other study. Scores based on PGC results were nominally associated with MDD in CONVERGE (Figure 1; Supplementary Tables S1–S3), accounting for ~0.1% of risk (Nagelkerke's pseudo-*R*^2^=7.46 × 10^−4^; *P*=0.02). Scores based on results for female-only yielded similar results (Nagelkerke's pseudo-*R*^2^=7.60 × 10^−4^; *P*=0.0141), whereas scores for recurrent MDD were most strongly associated overall (Nagelkerke's pseudo-*R*^2^=0.00201; *P*=6.56 × 10^−^^5^). Scores from CONVERGE were nominally associated with MDD status in the PGC data (Nagelkerke's pseudo-*R*^2^=6.08 × 10^−4^; *P*=6.66 × 10^−3^); these scores yielded similar results when considering female-only (Nagelkerke's pseudo-*R*^2^=0.00111; *P*=4.15 × 10^−3^), and recurrent MDD (Nagelkerke's pseudo-*R*^2^=9.13 × 10^−^^4^; *P*=2.02 × 10^−^^3^). However, only the results based on PGC-trained polygenic scores for recurrent MDD remained significant after correction for multiple testing (Supplementary Table S2).

We evaluated whether the observed fraction of results displaying the same direction of allelic effects across studies was significantly greater than expected by chance (that is, 50%) using binomial sign tests. Supplementary Table S4 gives the number of LD-independent SNPs considered, fraction of these SNPs displaying the same direction of effect in the other study and a one-sided binomial test *P*-value. For lifetime MDD, we observed the largest excess of same-direction effects in PGC for SNPs significant at *P*<0.2 in CONVERGE (50.7% binomial *P*=1 × 10^−3^); this finding remains significant after multiple-testing adjustment (Supplementary Table S4). For the reverse comparison, the largest excess of same-direction effects was observed for SNPS significant at *P*<0.2 in PGC (50.5% binomial *P*=0.016).

Overall, the greatest excess of same-direction effects in CONVERGE was observed for SNPs significant at *P*<0.1 in the PGC recurrent MDD analysis (51.1% binomial *P*=3.05 × 10^−5^); the fraction of same-direction effects in the PGC was largest in the female-only analysis, for SNPs significant at *P*<0.1 in CONVERGE (50.9% binomial *P*=1.11 × 10^−^^3^). Although statistically significant after correcting for multiple tests (Supplementary Table S4), the observed excess of same-direction effects represents only a very small deviation from expectation under the null hypothesis.

### Trans-ancestry genetic correlation

Table 2 displays the results of the trans-ancestry genetic correlation between East Asian and European populations. For lifetime MDD (*ρ*_g_=0.332, 95% confidence interval (CI): (0.270, 0.394)), this was both significantly greater than zero (*P*_ρg>0_=7.23 × 10^−^^26^) and significantly less than one (*P*_ρg<1_=1.40 × 10^−99^), indicating a partially shared polygenic basis of MDD risk between East Asians and Europeans. These findings remain significant after correction for multiple tests.

By comparison, recurrent MDD and females-only yielded slightly higher estimates of genetic correlation (Table 2). We compared these estimates by assuming an approximately normal distribution for *ρ*_g_ and obtaining a *Z*-score for the difference in values; these differences were found to be nominally significant for both recurrent MDD (*P*_one__-sided_=0.023) and females-only (*P*_one__-sided_=0.044). We followed up these results by calculating genetic effect correlation estimates based on comparisons of CONVERGE to *N*=60 random subsets of the PGC data (Supplementary Figure S1). Compared with lifetime MDD (*ρ*_g_*=*0.309; 95% CI: (0.290 0.327)), estimates of *ρ*_*g*_ were significantly higher for females-only (*ρ*_g_*=*0.372, 95% CI: (0.344,0.401); *t*(59)=7.41, *P*_one-sided_=2.69 × 10^−^^10^) and recurrent MDD (*ρ*_g_*=*0.375, 95% CI: (0.362,0.389); *t*(59)=15.29, *P*_one__-sided_=1.74 × 10^-22^).

To aid our interpretation of the cross-ancestry results, we derived analogous within-ancestry estimates for East Asians (*ρ*_g_*=*0.926, 95% CI: (0.967,0.967)) and Europeans (*ρ*_*g*_*=*0.807, 95% CI: (0.856,0.856); Supplementary Figure S2). Notably, within-ancestry analysis of East Asians yielded significantly greater estimates of *ρ*_g_ (*t*(56.45)=3.70, *P*_two-sided_=0.0005). However, as CONVERGE represents a single study, actual population differences are confounded here with those arising from ascertainment or heterogeneity in assessment methods and instruments among participating PGC studies.

### SNP-based meta-analyses

We observed the strongest overall evidence of association experiment-wide between SNPs upstream of gephyrin (*GPHN*) at 14q23.3 (rs9323497; log_10_BF=8.08) and lifetime MDD (Supplementary Figures S3 and S4). Associated SNPs show marked differences in allele frequencies between East Asian and European populations and opposing directions of allelic effect in CONVERGE and PGC (Supplementary Figure 4). This locus encodes a neuronal assembly protein that anchors glycine and GABAA receptors to the postsynaptic density in inhibitory neurons.^36^ Intriguingly, the gephryin region exhibits an unusual ‘yin-yang' haplotype structure reflecting strong positive selection related to recent, rapid human evolution,^37^ and has previously yielded suggestive evidence of association with depressive symptoms in the general population.^38^

A total of 10 independent associated SNPs (log_10_BF>5) were prioritized for replication (Supplementary Table S5; Supplementary Figure S4); of these, three were in or near *GPHN*, two represent previously reported associations in CONVERGE that did not replicate in PGC^6^ and one was the strongest reported association in the original PGC study.^22^ No single SNP in either the females-only or recurrent MDD analyses attained genome-wide significance (Supplementary Figure S3). From each of these analyses, seven independent associated SNPs were taken forward to the replication stage (Supplementary Tables S6 and S7).

We attempted to replicate these single-SNP associations in a collection of independent replication samples (4504 MDD cases and 7007 controls). For lifetime MDD, no single SNP yielded nominally significant evidence of association (*P*<0.05) in fixed-effects meta-analysis of these replication samples (Supplementary Table S5). Replication analyses also failed to generate replication support for SNP associations identified for females-only or recurrent MDD (Supplementary Tables S6 and S7). Regional association and forest plots for these SNPs are provided in the Supplementary Figures S4–S6.

For selected SNPs from the trans-ancestry meta-analyses, we assessed the significance of the observed fraction of SNPs showing the same direction of effect across discovery and replication phases; these fractions were 0.30 (*P*=0.9453), 0.286 (*P*=0.9375) and 0.571 (*P*=0.5) for lifetime, females-only and recurrent MDD, respectively.

### Gene-set enrichment analyses

We used DEPICT to investigate whether particular pathways or gene sets were enriched for associations with any of the phenotypic definitions considered. For SNPs significant at *P*<10^−^^5^ in meta-analyses of lifetime, females-only and recurrent MDD (29, 24 and 27 independent loci, respectively), no single pathway or gene set was significantly enriched, or contained more significant genes than expected by chance, after correction for multiple testing (*q*⩾0.20).

When we considered a more inclusive threshold (*P*<10^−4^), there were 167, 161 and 161 independent loci for lifetime, females-only and recurrent MDD, respectively. Following correction for multiple testing, only central nervous system neuron differentiation (GO:0021953) and axon cargo transport pathways (GO:0008088) were found to be significantly enriched (*q*<0.05) in the analysis of lifetime MDD. An additional 11 gene sets were suggestively enriched (*q*<0.20) and included several ontology terms related to neurodevelopmental processes (Supplementary Table S8). Finally, no tissue or cell types were enriched for associations with any definition of MDD (*q*⩾0.20), irrespective of the significance threshold applied.

## Discussion

We have conducted a large, trans-ancestry meta-analysis representing, to our knowledge, the first systematic effort to analyze European and Han Chinese studies of MDD. As expected, we identified a shared, common polygenic basis of MDD between these populations, as exemplified by an excess of same-direction allelic effects, significant polygenic risk score profiling results and modest estimates of genetic correlation.

We initially considered the simple hypothesis that disease-relevant SNP effects would have similar sizes and directions of effect across European and Han Chinese studies,^39^ without explicit consideration of population differences arising from genetic drift or divergent genetic architectures. Scores constructed from either PGC or CONVERGE results were significantly associated with lifetime MDD in the other study, albeit explaining a diminutive fraction of risk. However, it is commonly observed that polygenic prediction is generally poorer when ‘training' and ‘testing' data sets do not originate from a single ancestral population, likely attributable to differences in allele frequencies and patterns of LD.^20, 21^

Next, we applied the recently developed *popcorn* method^30^ to obtain estimates of the genetic effect correlation between these populations. Briefly, the genetic correlation is the correlation coefficient of per-allele SNP effect sizes across populations. We found that the genetic correlation of lifetime MDD was significantly different from both zero and one, suggesting that there is substantial but incomplete overlap in common SNP effects predisposing to MDD in Europe and China. Of particular interest, comparisons based on females-only or recurrent MDD, which better recapitulated the ascertainment strategy in CONVERGE, yielded significantly higher estimates of genetic correlation despite an attendant reduction in sample size.

Given the extensive heterogeneity of MDD, and an expected and demonstrable loss of power arising from between-study differences in ancestry and ascertainment, our limited success in identifying novel, replicable evidence of genome-wide significant association is perhaps unsurprising. It is well understood that a trait's heritability—and by extension, a shared polygenic liability—is a less important determinant of successful identification of relevant associations than its underlying genetic architecture. Considering the relatively low genetic correlations reported here, we might expect an attenuation of statistical power to detect individual variants, that is, as compared with a similarly sized studies of the same ancestry. A concomitant, statistically significant enrichment of biologically relevant gene sets is taken as an additional support for this interpretation.

### Limitations

First, the absence of replicable associations with MDD in ancestrally diverse populations precluded more pointed comparisons of specific genetic effects.

Our attempts to reduce the heterogeneity of MDD, namely by focusing on two particular subtypes of illness, should be regarded as preliminary. Furthermore, questions pertaining to both screening and ascertainment of controls were not addressed in the current study, and could have reduced our power to detect relevant variation. We expect that with larger sample sizes, future studies will be sufficiently powered to address these issues.

Finally, by having conducted multiple separate analyses for females-only and recurrent MDD, we increased the multiple-testing burden. As these do not represent completely independent analyses, we have not corrected exhaustively for the total number of tests performed.

## Conclusions

We have demonstrated a common polygenic basis of MDD that is partially shared between European and Han Chinese populations. Importantly, our findings appear to reinforce the idea that subtyping of MDD may yield additional insight into its etiology.^40^ Striking an advantageous balance between phenotypically more homogeneous definitions of illness and sample size represents an ongoing and nuanced challenge for genetic studies of MDD.

## Figures and Tables

**Table 1:** Sample sizes by participating study site in discovery and replication phases

*Phase*	*Study*	*Ancestry*	*No. of controls*	*No. of cases*	*No. of recurrent*
Discovery	Bonn Mannheim	EUR	1072 (48.3)	588 (64.1)	408 (66.7)
	GenRED1	EUR	1097 (42.7)	1020 (70.8)	1020 (70.8)
	GSK MPIP	EUR	859 (67.6)	861 (67.4)	831 (67.3)
	MPIP MARS 650	EUR	539 (54.5)	373 (55.2)	128 (59.4)
	NTR/NESDA	EUR	1727 (62.0)	1685 (69.2)	834 (71.2)
	QIMR I317	EUR	960 (55.2)	1017 (61.0)	524 (65.6)
	QIMR I610	EUR	748 (61.9)	433 (72.3)	250 (72.4)
	RADIANT	EUR	1549 (54.2)	1903 (70.5)	1441 (70.8)
	RADIANT—Germany	EUR	217 (53.9)	327 (65.7)	254 (70.5)
	STAR*D	EUR	447 (43.8)	1240 (58.6)	912 (59.1)
	CONVERGE^a^	EAS	5220	5282	5282
Replication^b^	Edinburgh	EUR	285 (48.8)	372 (59.4)	—
	DepGenesNetwork	EUR	470 (62.6)	471 (77.5)	471 (77.5)
	GenRED2	EUR	474 (48.9)	830 (82.8)	830 (82.8)
	Harvard i2b2	EUR	1067 (50.1)	806 (66.9)	806 (66.9)
	Janssen	EUR	1380 (60.5)	466 (68.2)	466 (68.2)
	QIMR COEX	EUR	526 (57.6)	565 (71.5)	—
	RADIANT—Irish cases	EUR	340 (52.4)	109 (82.6)	109 (82.6)
	RADIANT—US cases	EUR	378 (51.9)	223 (78.5)	223 (78.5)
	RADIANT—Denmark cases	EUR	516 (40.7)	133 (69.9)	133 (69.9)
	SHIP-LEGEND	EUR	1087 (44.0)	366 (67.8)	—
	SHIP-TREND-0	EUR	484 (44.6)	163 (71.8)	—
Totals	Discovery		14 435 (71.3)	14 729 (78.4)	11 884 (82.2)
	Replication		7007 (51.6)	4504 (72.3)	2572 (75.8)

Abbreviations: EAS, East Asian; EUR, European.

Numbers of female subjects are displayed parenthetically.

a All cases and controls were female

.

b See note in Materials and methods section.

**Table 2:** Trans-ancestry genetic correlations between East Asian and European MDD subtypes

*Trait*^a^	ρ_*g*_^b^	P_*ρg>0*_	P_*ρg<1*_
Lifetime MDD	0.332 (0.270, 0.394)	7.23 × 10^−^^26^	1.40 × 10^−99^
Female-only MDD	0.402 (0.326, 0.477)	2.04 × 10^−25^	2.59 × 10^−54^
Recurrent MDD	0.410 (0.343, 0.477)	5.40 × 10^−33^	2.23 × 10^−^^66^

Abbreviation: MDD, major depressive disorder.

a European prevalences of lifetime, females-only and recurrent MDD were assumed to be 0.15, 0.20 and 0.105, respectively; prevalence of recurrent MDD among Chinese women was assumed to be 0.08.

b Estimates of *ρ*_g_ are displayed with corresponding 95% confidence intervals.
