# Supplementary material for: Genetic effects influencing risk for major depressive disorder in China and Europe
Source: Transl Psychiatry. 2017 Mar 28;7(3):e1074–. doi: 10.1038/tp.2016.292 (PMC5404611; doi:10.1038/tp.2016.292)
Supplement: Supplementary Information [file tp2016292x1.docx]

**Supplemental Information**

# **Descriptions of Participating Replication Studies**

## *Generation Scotland:Scotland Family Health Study*

GS:SFHS is a population-based sample designed to identify the genetic causes of common complex diseases. The complete study protocol and other summary characteristics have been described in detail elsewhere [(1–3)](https://paperpile.com/c/Tlfggg/JhZh+WzBM+0TXr). Briefly, participants were recruited from primary care general medical practitioner registries across Scotland. In order to minimize ascertainment bias, MDD cases were neither actively recruited nor used to recruit related affected persons. Recruitment was initially limited to Glasgow and Tayside and subsequently extended to include Ayrshire, Arran and Northeast Scotland. Relatives of recruited individuals could come from any location. A total of 20 198 invitees and their relatives volunteered and completed all aspects of the extensive phenotyping, which included pre-clinic questionnaires and a two-hour face-to-face assessment. Participants were informed the purpose of the study was to study the health of the Scottish population and gave written consent, after having an opportunity to discuss the project, and before any data or samples were collected.

## *Genetics of Recurrent Early-Onset Depression Phase II*

The second phase of GenRED was included as a replication sample (the first phase was a discovery sample in the original PGC study)[(4)](https://paperpile.com/c/Tlfggg/evY0). GenRED2 included new cases meeting the same criteria as in GenRED (see above), plus new controls. Dr. Janet Sobell (University of Southern California) contributed 287 post-QC controls from the Mayo DNA Bank which consists of long-term, community medicine patients (Mayo Clinic, Rochester, MN) who were undergoing venipuncture for any reason. Consenting individuals ages 45 and above completed a brief demographic and psychiatric screening questionnaire. Extensive medical records were screened for evidence of psychiatric illness. Individuals were excluded if they were judged likely to have had a mood or psychotic disorder on the basis of a review of medical records, taking into account the recorded diagnoses and treatment in each case (including major disorders as well as possible proxies for a mood disorder in older nomenclature such as adjustment disorders, depression NOS, anxiety state, etc.). The final subset was selected to roughly match the proportions of self-reported ancestry in the GenRED 1+2 sample. Drs. Carlos Pato and James Knowles contributed 187 post-QC controls from the Genomic Psychiatry Consortium, an ongoing study of schizophrenia and bipolar disorder.

Controls were recruited opportunistically in the Los Angeles area and screened with a self-report questionnaire. We selected individuals who reported European ancestry in all grandparents and answered negatively to five screening questions for lifetime bipolar disorder, five for lifetime schizophrenia, and four for lifetime MDD. Note that these 1 305 subjects were genotyped at the same lab (Centrillion Biosciences, Mountain View, CA) and with the same GWAS array (Illumina Omni1-Quad) as the Depression Genes and Networks sample (below) collected by a subset of the GenRED investigators, so that these samples were combined for the PGC replication analysis.

## *Depression Genes and Networks*

This sample was genotyped and analyzed in combination with the GenRED2 sample (above). A survey research company (Knowledge Networks, Menlo Park, CA) recruited the 471 post-QC recurrent MDD cases and 470 never-depressed controls from participants in an online survey panel that is recruited on an ongoing basis using random digit dialing of nationally representative US households. (Note that the same panel was used to recruit the Molecular Genetics of Schizophrenia control sample for the NIMH repository, but individuals who were invited to be screened for MGS and who were still members of the panel were not invited to be screened for DGN). Online screening was carried out using the CIDI-SF depression and alcohol and substance dependence modules; prospective controls were selected who reported two or more episodes meeting criteria for MDD but denied lifetime substance dependence, while prospective controls denied ever having two or more weeks of depressed mood or anhedonia and two or more other MDD criteria outside of acute bereavement. These individuals were then interviewed (SCID) and individuals not meeting the initial eligibility criteria (based on final review by the site PI) were excluded. Note that these cases all reported recurrent MDD, but were not required to meet the additional criteria required for the GenRED project.

## *Harvard i2b2*

Details of the i2b2 study are detailed elsewhere[(5)](https://paperpile.com/c/Tlfggg/IGIj). Briefly, cases were identified using the Partners Healthcare electronic medical record (EMR). Using data available in the EMR, patients who met the following inclusion criteria were identified: a minimum of one ICD-9 code for MDD (ICD9 296.2x, 296.3x), at least one antidepressant prescription, and at least three outpatient psychiatry visit notes. Patients with both a past antidepressant prescription and at least two electroconvulsive therapy procedures (CPT: 90870, 90871) within one week were also included. Exclusion criteria included a past history of bipolar disorder (ICD-9: 296.1x, 296.4x-296.9x), dementia/delirium (ICD-9: 290.x), psychotic disorder (ICD-9: 295.x, 298.x), or past bone marrow transplant. De-identified, discarded blood specimens were obtained for subjects meeting these criteria who had presented for routine blood draw.

Genotyping was restricted to individuals who identified as having European ancestry. The Partners Institutional Review Board approved all aspects of this study. Controls were identified from a healthy volunteer sample, age 18-35, participating in the MGH Brain Genomics Superstruct Project and were screened to have no history of psychiatric or neurologic illness or treatment with psychoactive drugs.

## *Janssen*

The Janssen cohort included unrelated patients with major depressive disorder (*N*=545) from 3 clinical trials (NCT00044681, NCT01640080, and NCT01627782, Supplementary Table 1) conducted by Janssen Research & Development, LLC. The study inclusion/exclusion criteria have been described in detail in prior publications.(Rapaport et al. 2006, Singh et al. 2015) Briefly, all patients met DSM-IV criteria of Major Depressive Disorder and failed to respond to at least one antidepressant in the current episode. Additional detailed descriptions of these clinical trials can be found at ClinicalTrials.gov. Genetic sampling is either optional or mandatory, and all patients providing genetic samples consented to the genetic testing. Samples were genotyped using HumanOmni5Exome (Illuminia, Inc., San Diego, CA). Only patients of European ancestry with matching controls (*N*=466) were included in this study.

Controls were obtained from dbGAP, and originated from the Genome-Wide Association Study of Parkinson Disease: Genes and Environment study (phs000196.v1.p1). Briefly, all subjects were white and provided informed consent; inclusion criteria were age of 20 years or older, and no neurologic disorder at enrollment, by self report or exam, including Alzheimer's, Bipolar, Multiple Sclerosis, Amyotrophic Lateral Sclerosis, Ataxia, Dystonia, Parkinson's, Autism, Dementia, Epilepsy, Stroke and Schizophrenia.

## *QIMR COEX (MDD2000)*

The original MDD2000 study was a case-only study with MDD cases recruited by three research groups: Queensland Institute for Medical Research (QIMR), VU University Amsterdam and VU Medical Centre. The published study also included additional cases and controls from QIMR referred to as the I317, I370 and I610 sets (named to reflect the genotyping platforms, Illumina 317K, 370K and 610K)[(6)](https://paperpile.com/c/Tlfggg/KTcM). The current set represents additional subjects genotyped using the Illumina HumanCoreExome array(Cuellar-Partida et al. 2015), which were not included in the original PGC analysis [(7)](https://paperpile.com/c/Tlfggg/yGaA).

Briefly, the diagnosis of MDD was per DSM-IV criteria (consensus between two psychiatrists) using data from direct interview by a psychiatrist (SADS-L) supplemented by clinical interviews and medical record review. Particular attention was paid to exclude cases with bipolar spectrum disorders. Unrelated controls were selected as genotyped individuals from families in which no individuals qualified for diagnoses of MDD or anxiety disorders. All subjects are of North European ancestry and provided written informed consent under study protocols approved by the QIMR Human Research Ethics Committee.

## *RADIANT Studies (US, Irish, and Danish cases)*

Cases with recurrent unipolar depression were recruited from eight clinical sites (Aarhus, Denmark; Bonn, Germany; Dublin; Lausanne, Switzerland; St. Louis; London; Cardiff, United Kingdom; and Birmingham, United Kingdom), and were all of European ancestry. Eligibility criteria were as follows: age ≥18 years and experiencing ≥2 depressive episodes of at least moderate severity separated by at least 2 months of remission as defined by DSM-IV or ICD-10-DCR criteria. Subjects were excluded if having ever fulfilled criteria for mania, hypomania, or schizophrenia or having experienced psychotic symptoms that were mood incongruent or present when there was no evidence of a mood disturbance. Other exclusion criteria were intravenous drug dependency and depression occurring solely in relation to alcohol use. All participants gave written informed consent for participation in the study. Further details regarding clinical assessment have been described elsewhere [(8)](https://paperpile.com/c/Tlfggg/uCbr).

Control subjects for the US study were recruited from the Clinical Brain Disorders Branch of the NIMH ‘Sibling Study’. All subjects gave informed consent and were of European ancestry, and were genotyped at the NIMH. Control subjects for the Danish study were collected at the University of Aarhus, and included 500 medical students of Danish parentage for at least three generations. All subjects gave written informed consent and the Danish Data Protection Agency and the ethics committees of Denmark approved the human subjects protocol. Control subjects for the Irish study were collected as part of a amyolateral sclerosis study conducted in Dublin, Ireland [(9)](https://paperpile.com/c/Tlfggg/9R4d). All subjects reported no personal or family history of neurological disease and were of self-reported Irish Caucasian ethnicity for at least 3 generations.

## *SHIP-LEGEND*

Data from the Study of Health in Pomerania (SHIP) were used [(10)](https://paperpile.com/c/Tlfggg/NfMT). The target population was comprised of adult German residents in northeastern Germany living in 3 cities and 29 communities, with a total population of 212 157. A two-stage stratified cluster sample of adults aged 20-79 years (baseline) was randomly drawn from local population registries. The net sample (without migrated or deceased persons) comprised 6 267 eligible subjects of whom 4 308 Caucasian subjects participated at baseline SHIP-0 between 1997 and 2001. Follow-up examination (SHIP-1) was conducted 5 years after baseline and included 3 300 subjects. In 2007, the “Life-Events and Gene-Environment Interaction in Depression” (LEGEND) study was started based on SHIP [(11)](https://paperpile.com/c/Tlfggg/FaUn). The lifetime diagnosis of MDD was assessed with the Munich-Composite International Diagnostic Interview (M-CIDI). The M-CIDI is a standardized fully structured instrument for assessing psychiatric disorders over the life span according to DSM-IV criteria. The computerized version of the interview was used by clinically experienced psychologists in a face-to-face situation. All interviewers had undergone intensive and continuous training in the diagnostic procedures. SHIP and LEGEND were approved by the local Institutional Review Board and conformed to the principles of the Declaration of Helsinki.

The SHIP sample was genotyped using the Affymetrix Human SNP Array 6.0. Genotypes were determined using the Birdseed2 clustering algorithm. For quality control purposes, several control samples where added. Finally, all arrays had a sample call-rate>92%. The overall genotyping efficiency of the GWA was 98.55%.

## *SHIP-TREND*

From 2008 to 2012 a new independent sample called SHIP-Trend-0 (*N*=4,420) in the same area under the same recruitment strategies was drawn in 2008 and similar examinations were undertaken [(10)](https://paperpile.com/c/Tlfggg/NfMT). The lifetime diagnosis of MDD (DSM-IV) was assessed with the M-CIDI chapter for MDD by trained examiners.

Genotyping of a subset of the SHIP-TREND-0 subjects was performed using the Illumina Human Omni 2.5 array. Hybridization of genomic DNA was done in accordance with the manufacturer’s standard recommendations at the Helmholtz Zentrum München. Genotypes were determined using the GenomeStudio Genotyping Module v1.0 (GenCall algorithm). All 986 arrays had a genotyping rate of at least 94%. The overall genotyping efficiency of the GWA was 99.67%.

# **Supplemental Figures**

## *Supplemental Figure S1. Distributions of Trans-ancestry Genetic Correlation.* Genetic correlation (*⍴_g_*) between CONVERGE and *N*=60 random PGC subsets was calculated using POPCORN. Polygon widths are proportional to the number of replicates with a given value; horizontal lines indicate median estimates.


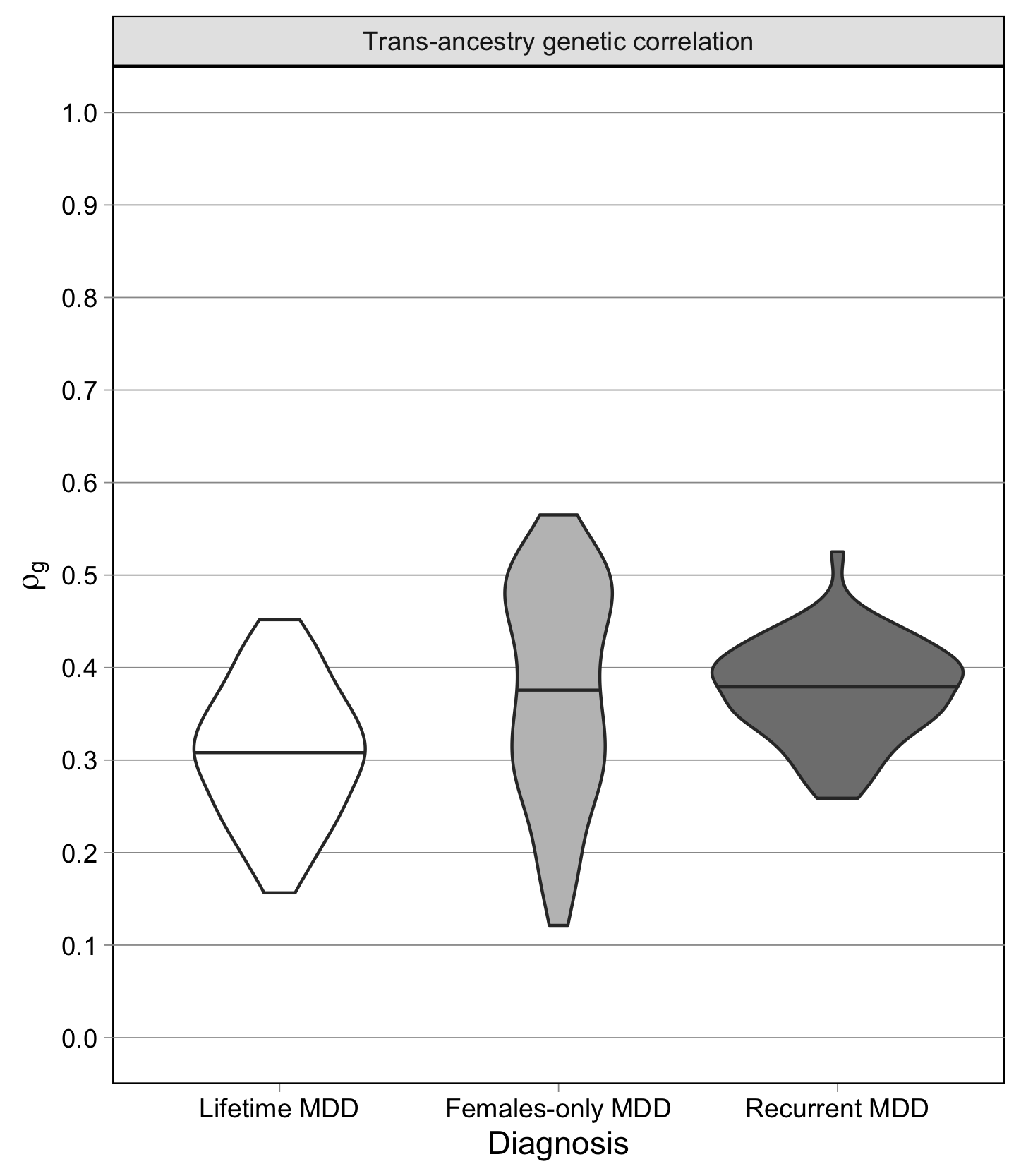


## *Supplemental Figure S2. Distributions of Within-Ancestry Genetic Correlations.* Genetic correlations (*⍴_g_*) between random split-halves of CONVERGE (*N*=30) or the PGC data (*N*=30) were obtained using POPCORN. Polygon widths are proportional to the number of replicates of a given value; horizontal lines indicate median estimates.


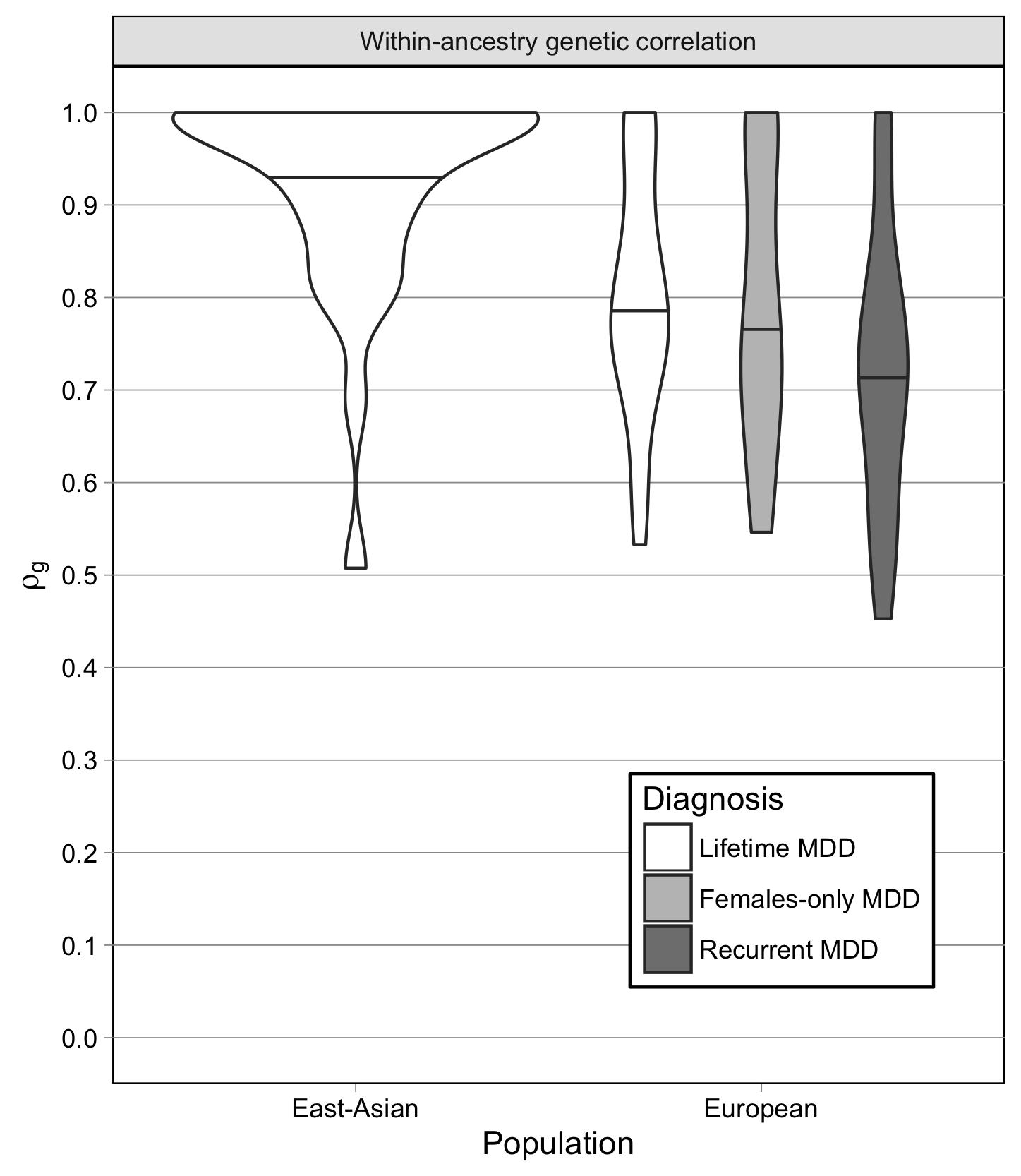


## *Supplemental Figure S3***.** Manhattan plots for trans-ancestry meta-analyses of Lifetime MDD, females-only MDD, and recurrent MDD. Red and blue lines indciate thresholds for genome-wide significance (log_10_BF>7) and replication follow-up (log_10_BF>5). For regions significant at the latter, the most significant “independent” SNP within a 500kb region is displayed as a blue diamond; nearby SNPs in linkage disequilibrium (*r*^2^ > 0.1) are highlighted.


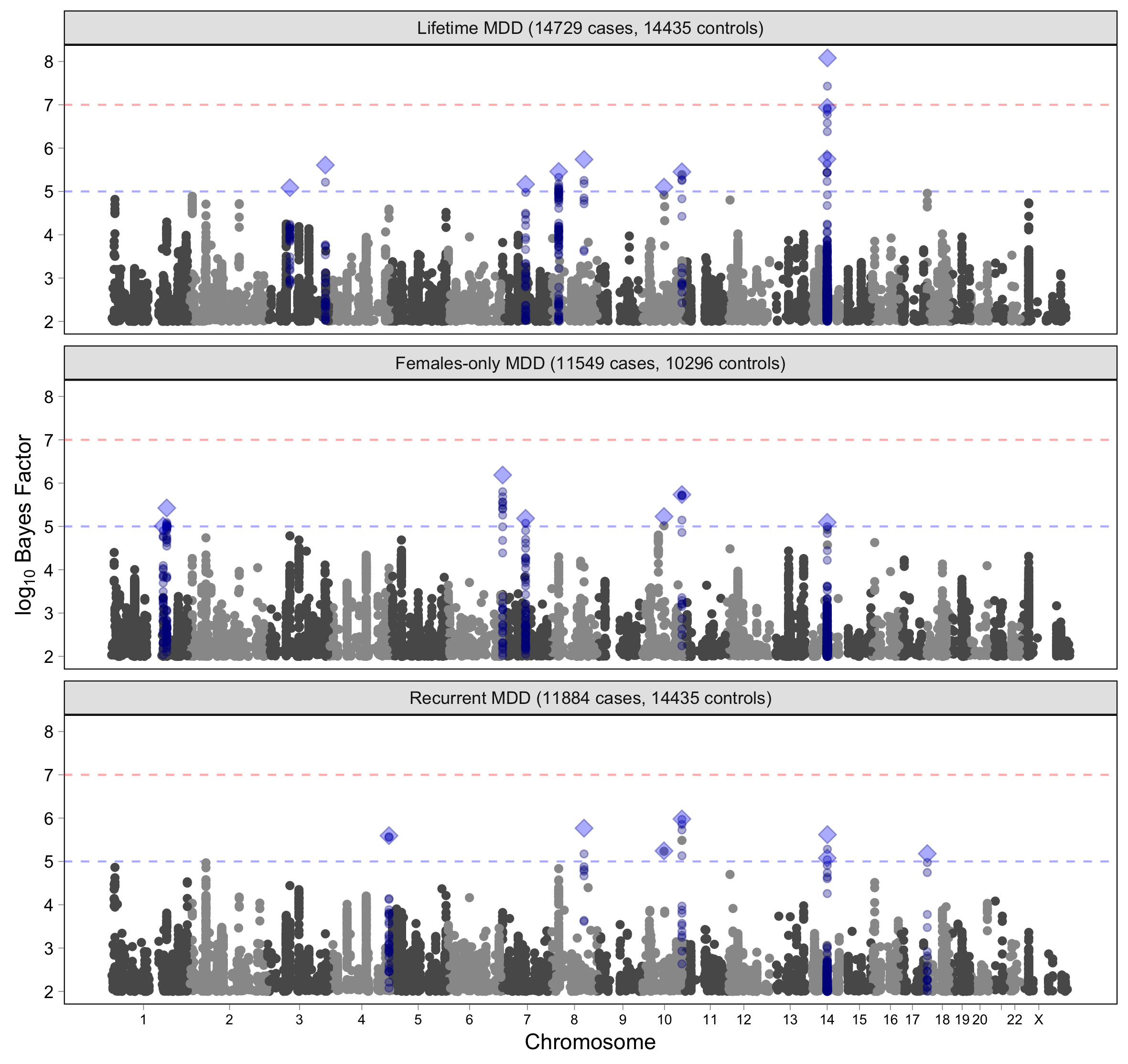


## *Supplemental Figure S4. Regional Association and Forest Plots for Lifetime MDD***.** ***(upper)*** Regional association plot created using LocusZoom [(12)](https://paperpile.com/c/Tlfggg/xcP5). LD of each SNP with the “index” SNP, displayed as a large purple diamond, is indicated by its color. ***(lower)*** *Study* abbreviations “conv”, “pgc1”, and “repli” are CONVERGE, PGC, and replication sample; *HetP* is the *P*-value for Cochran’s test of heterogeneity.


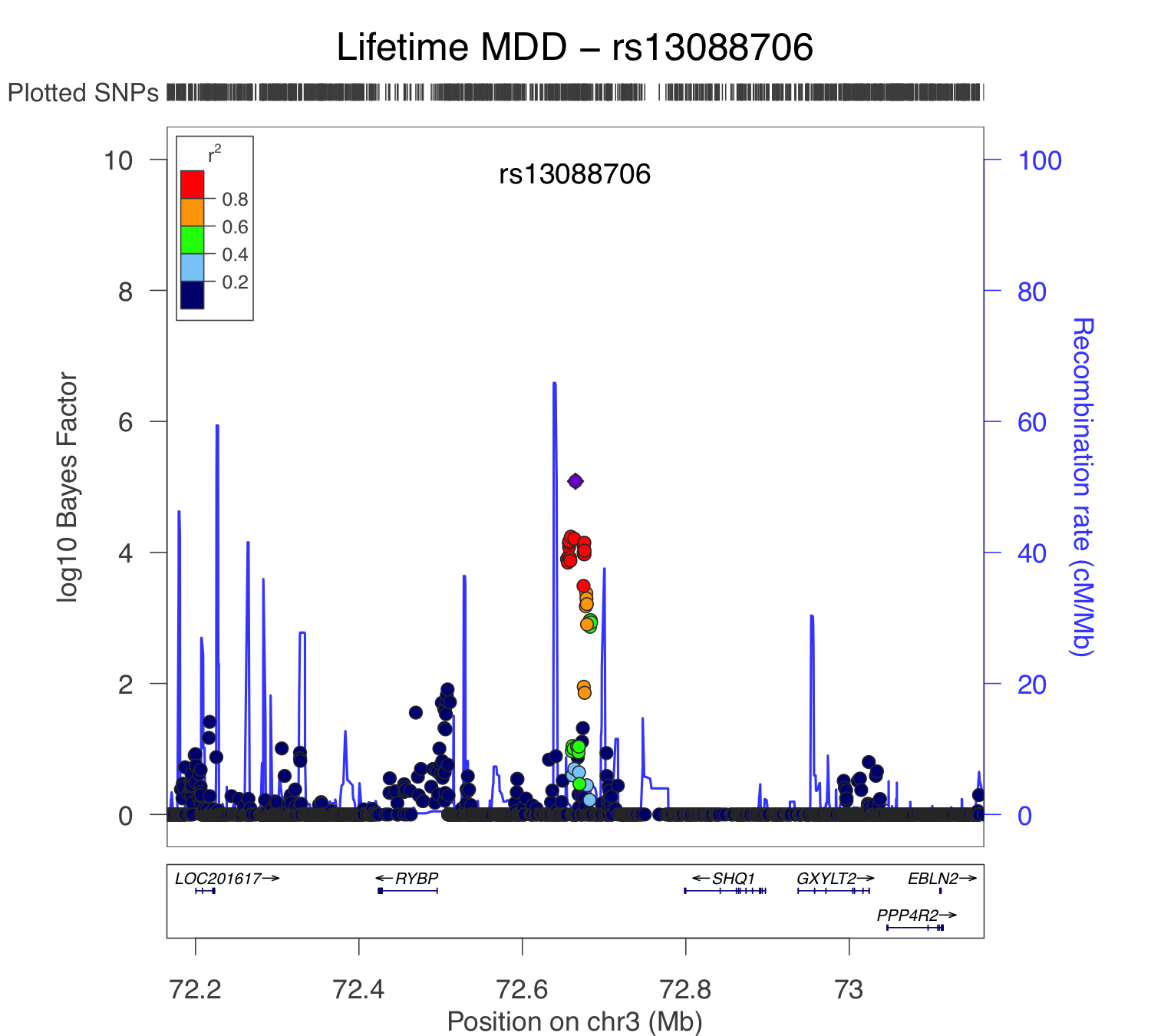

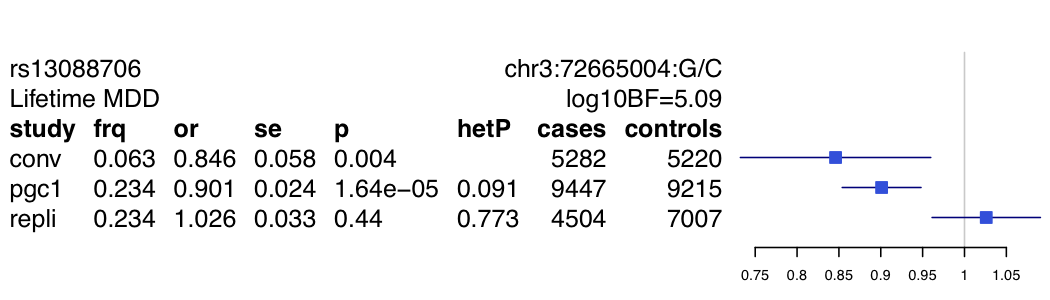

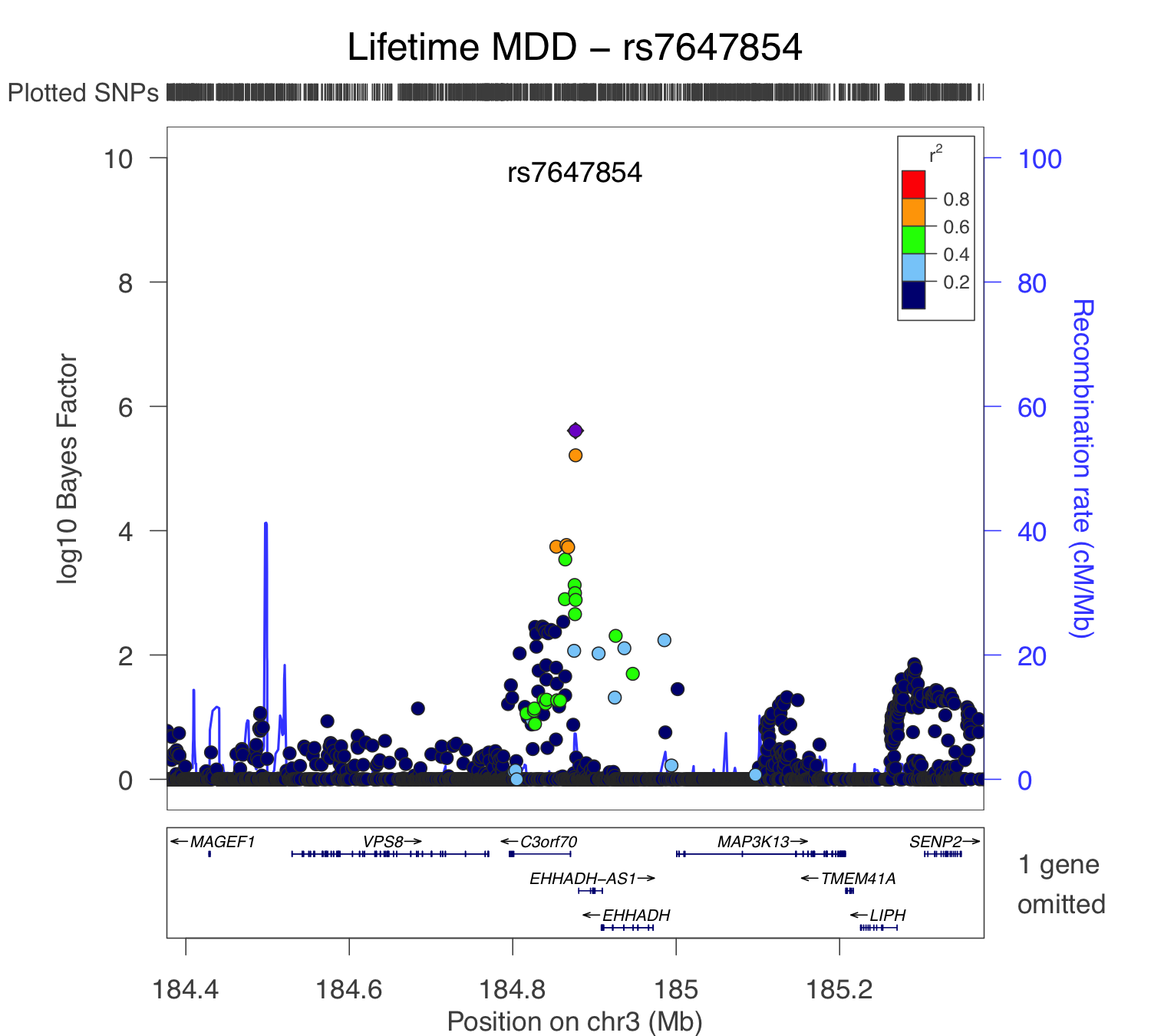

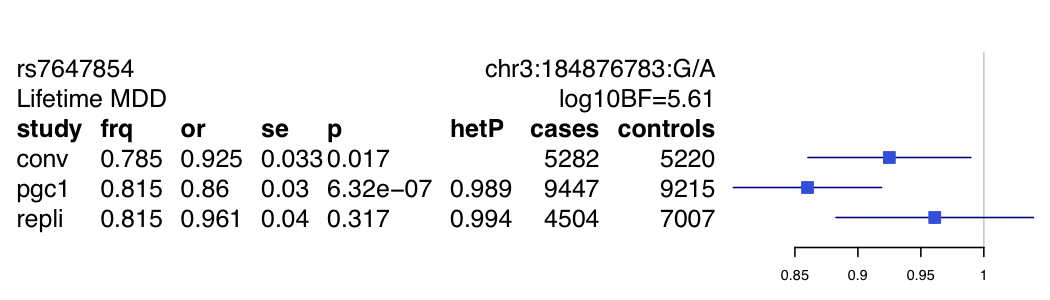

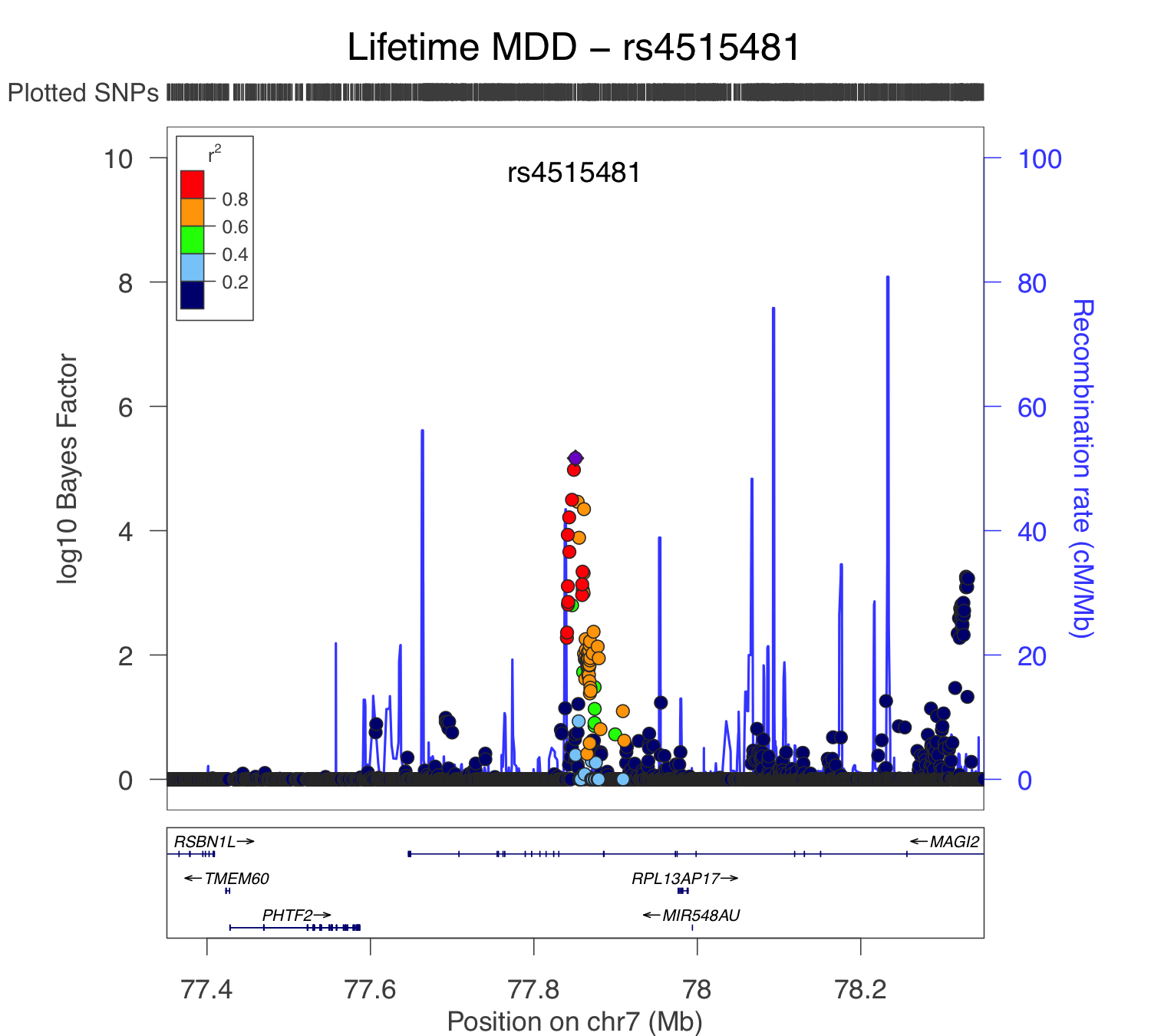

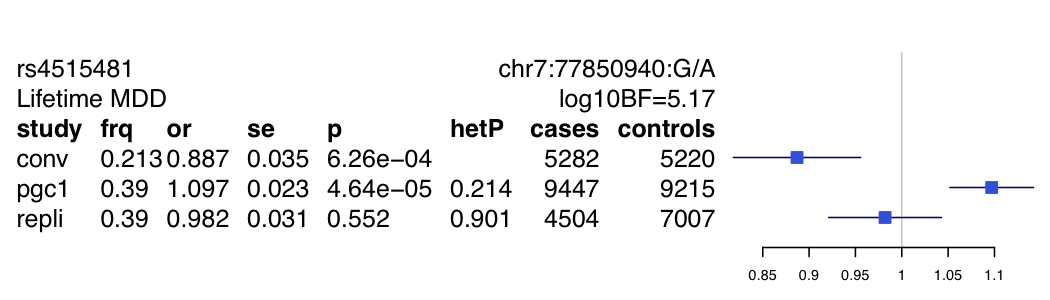

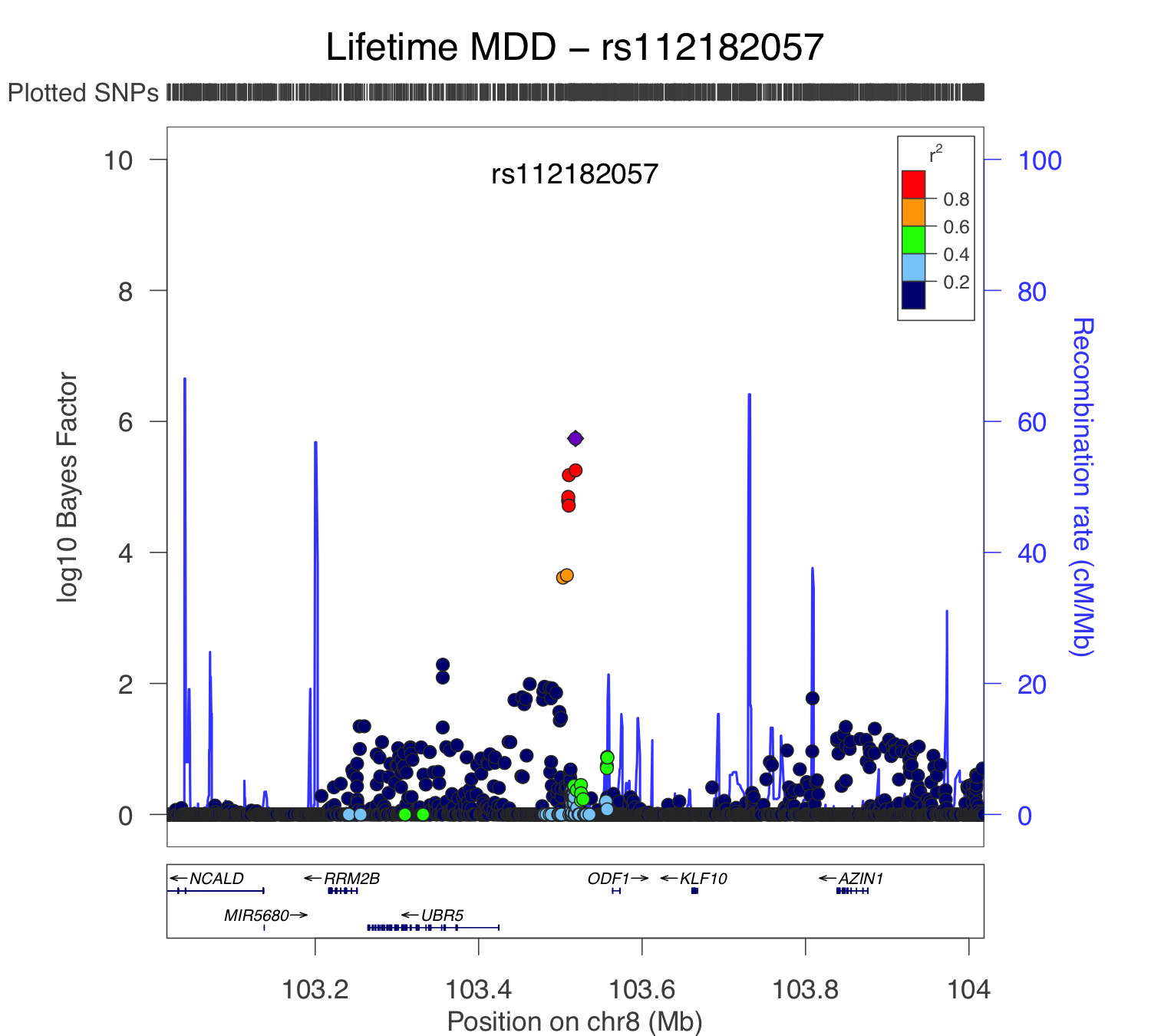

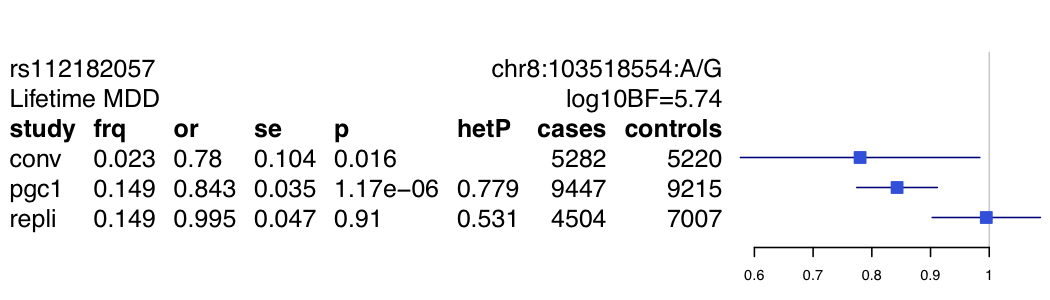

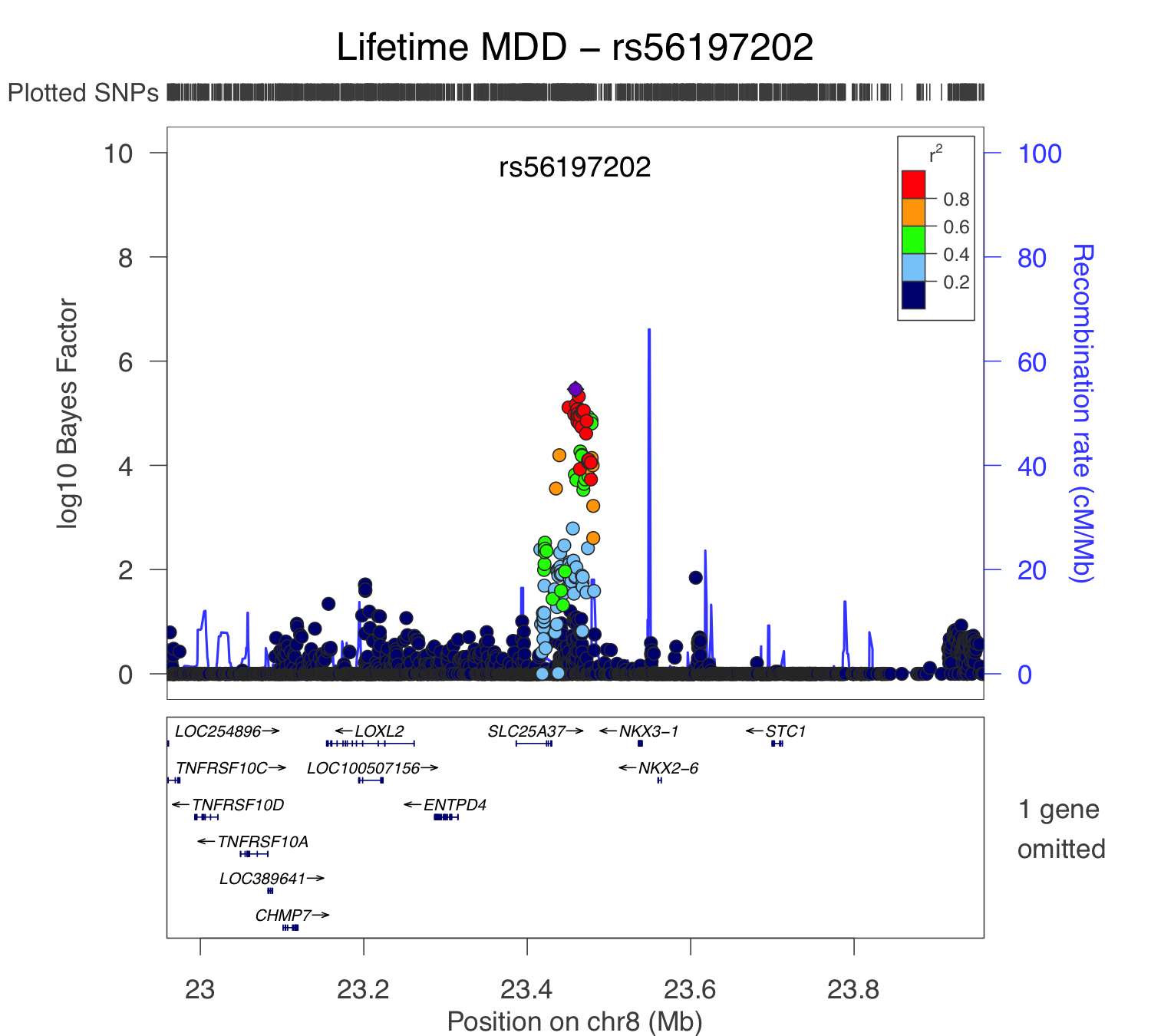

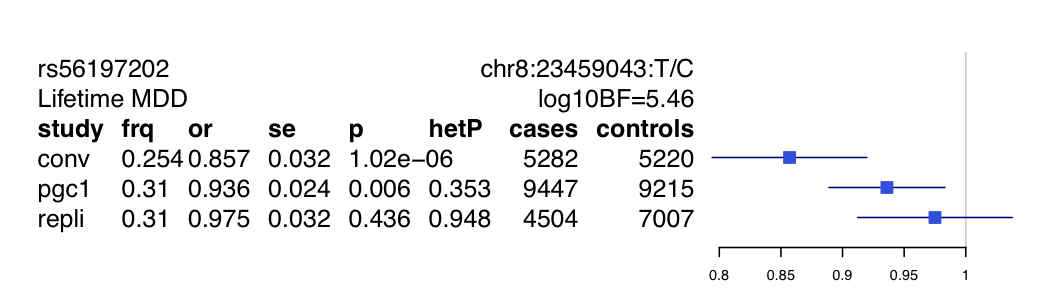

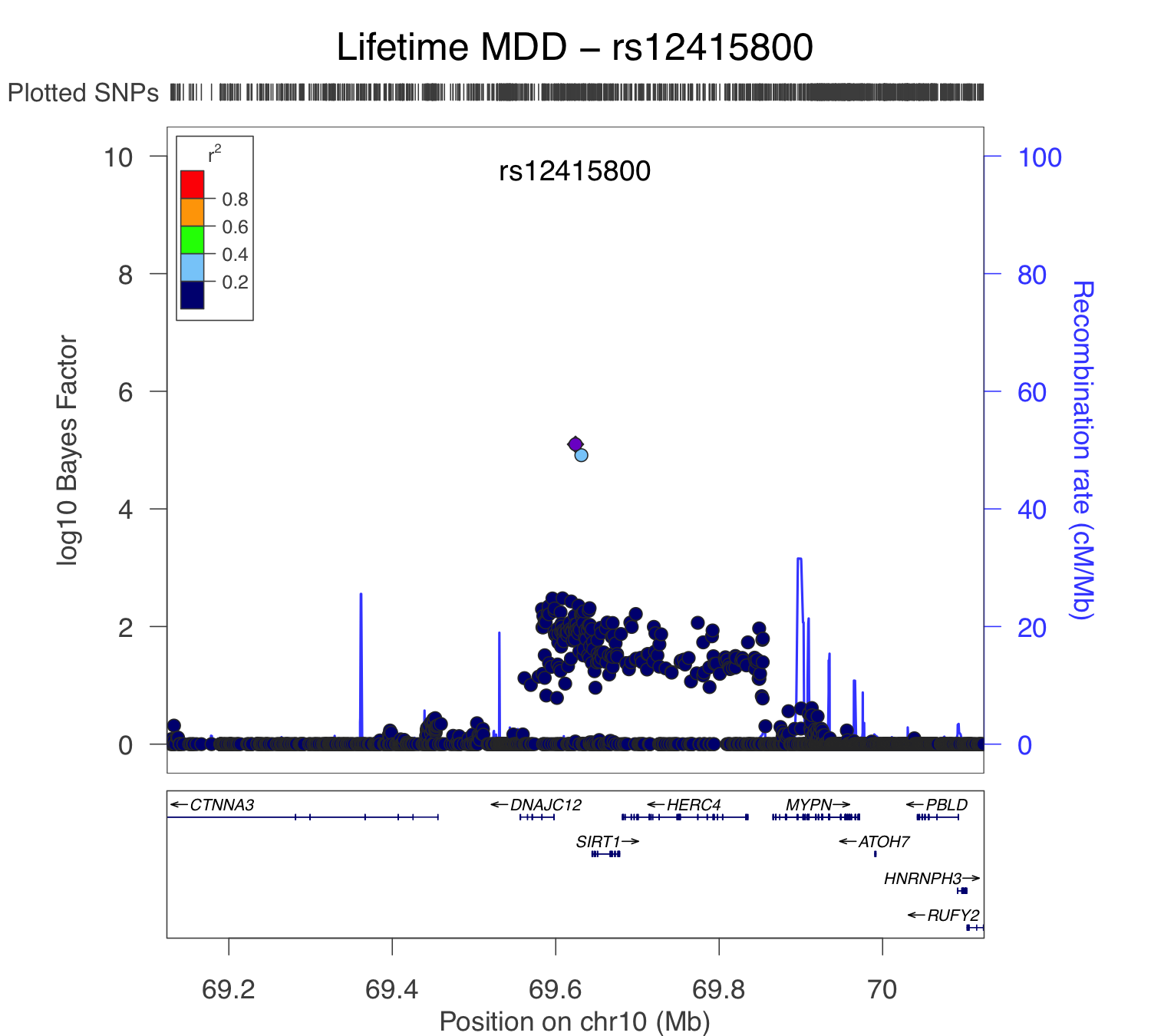

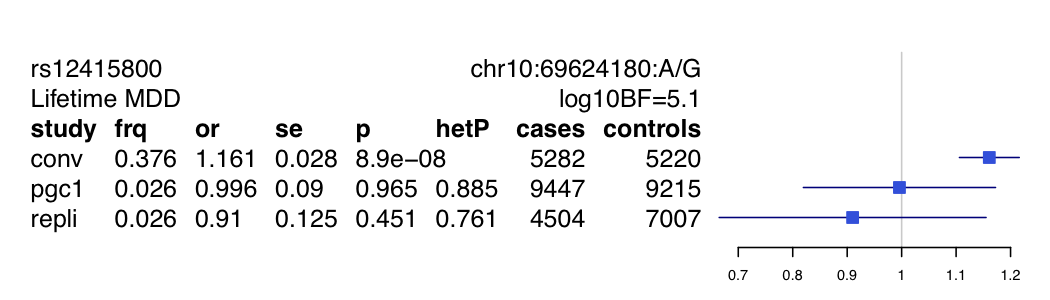

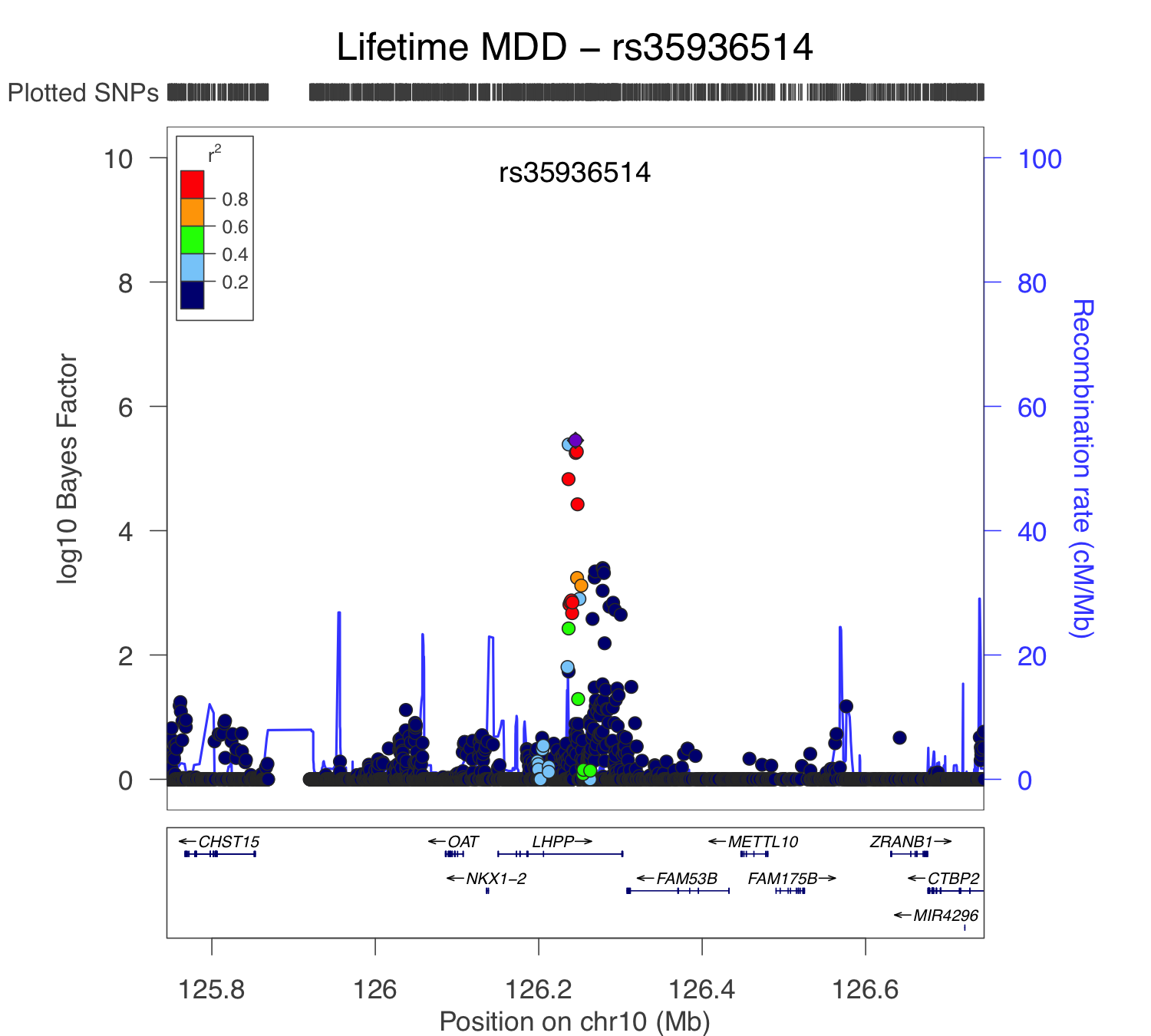

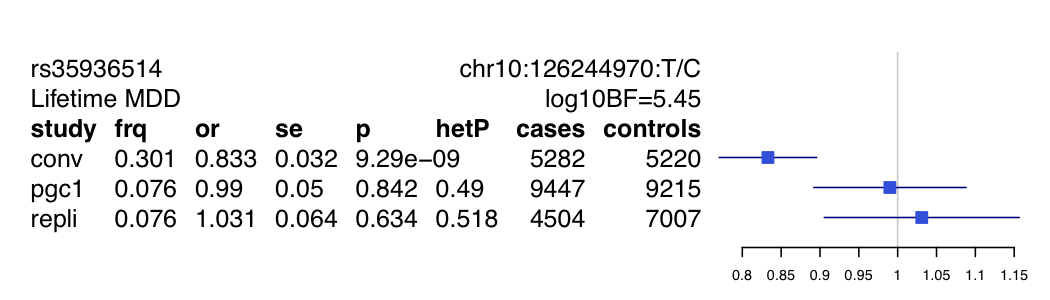

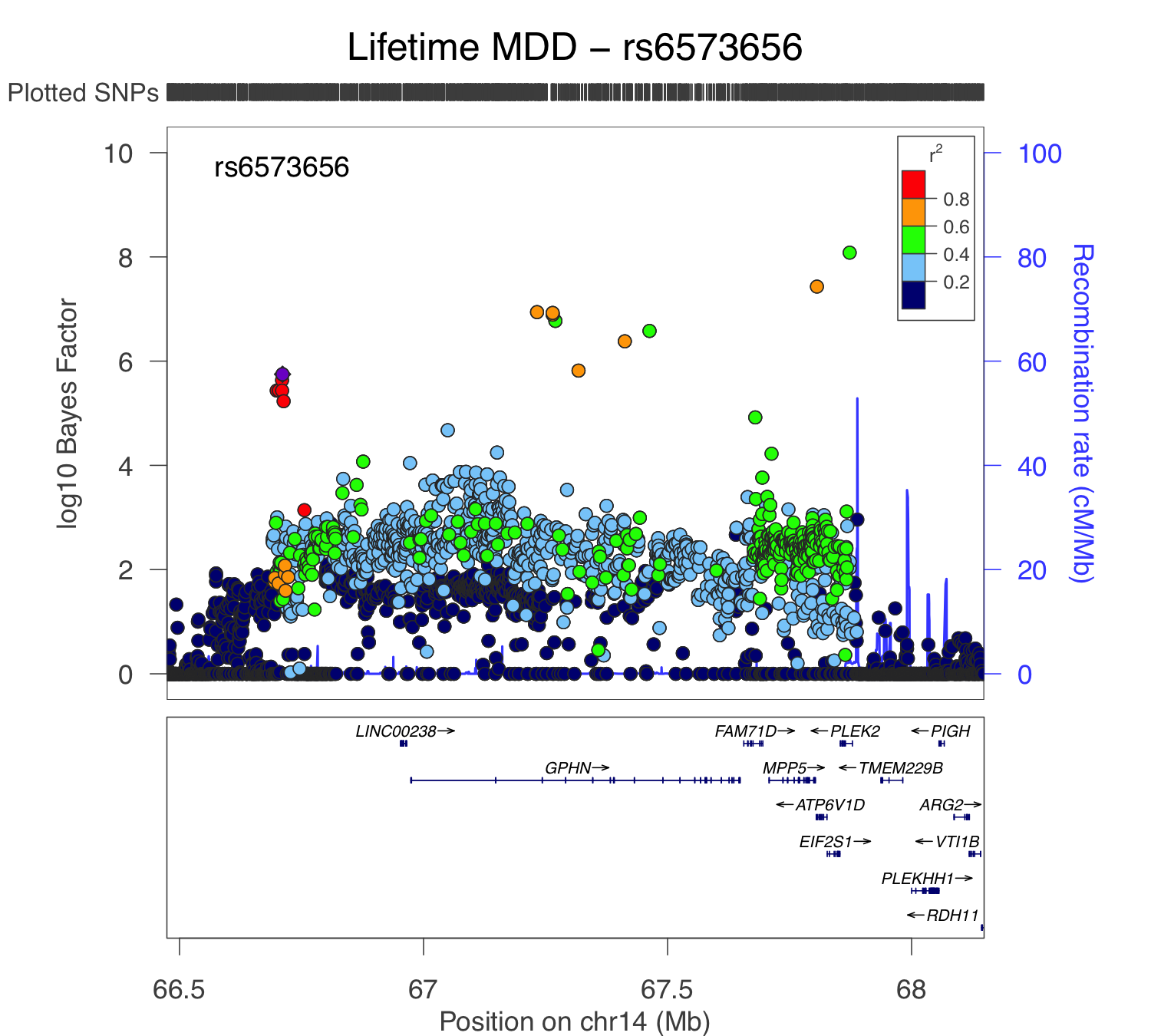

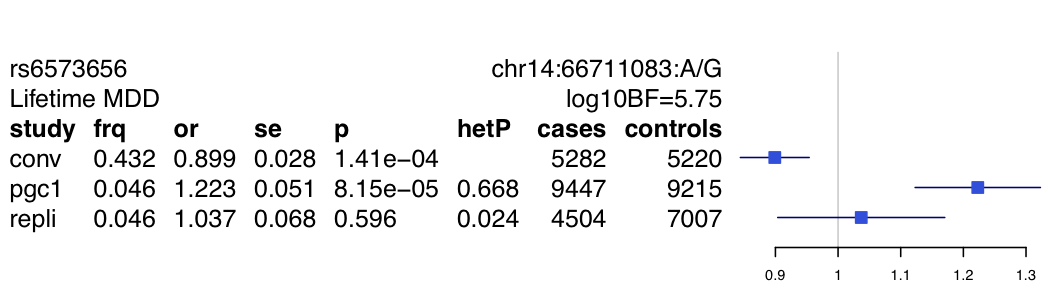

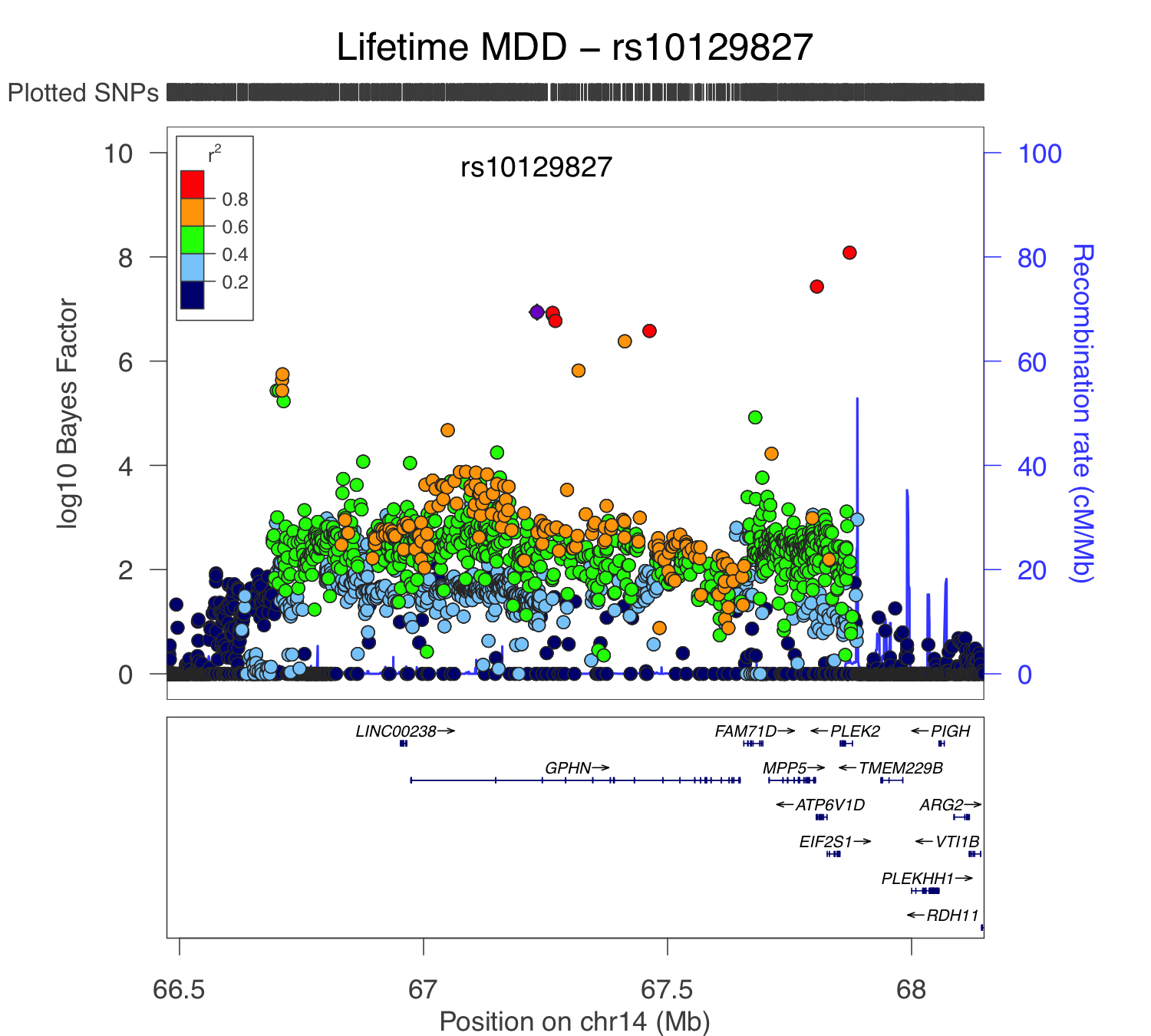

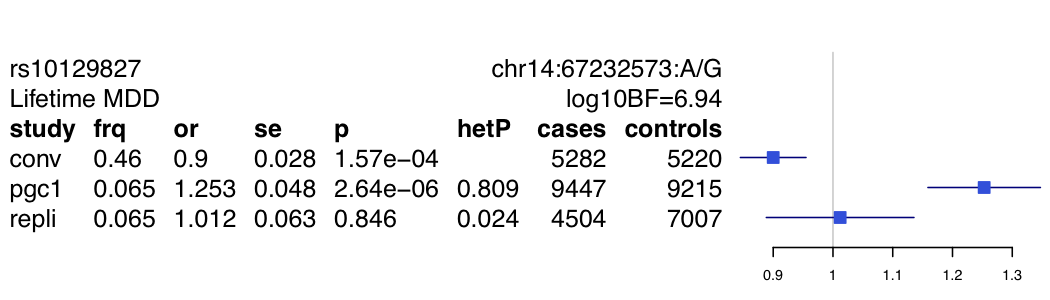

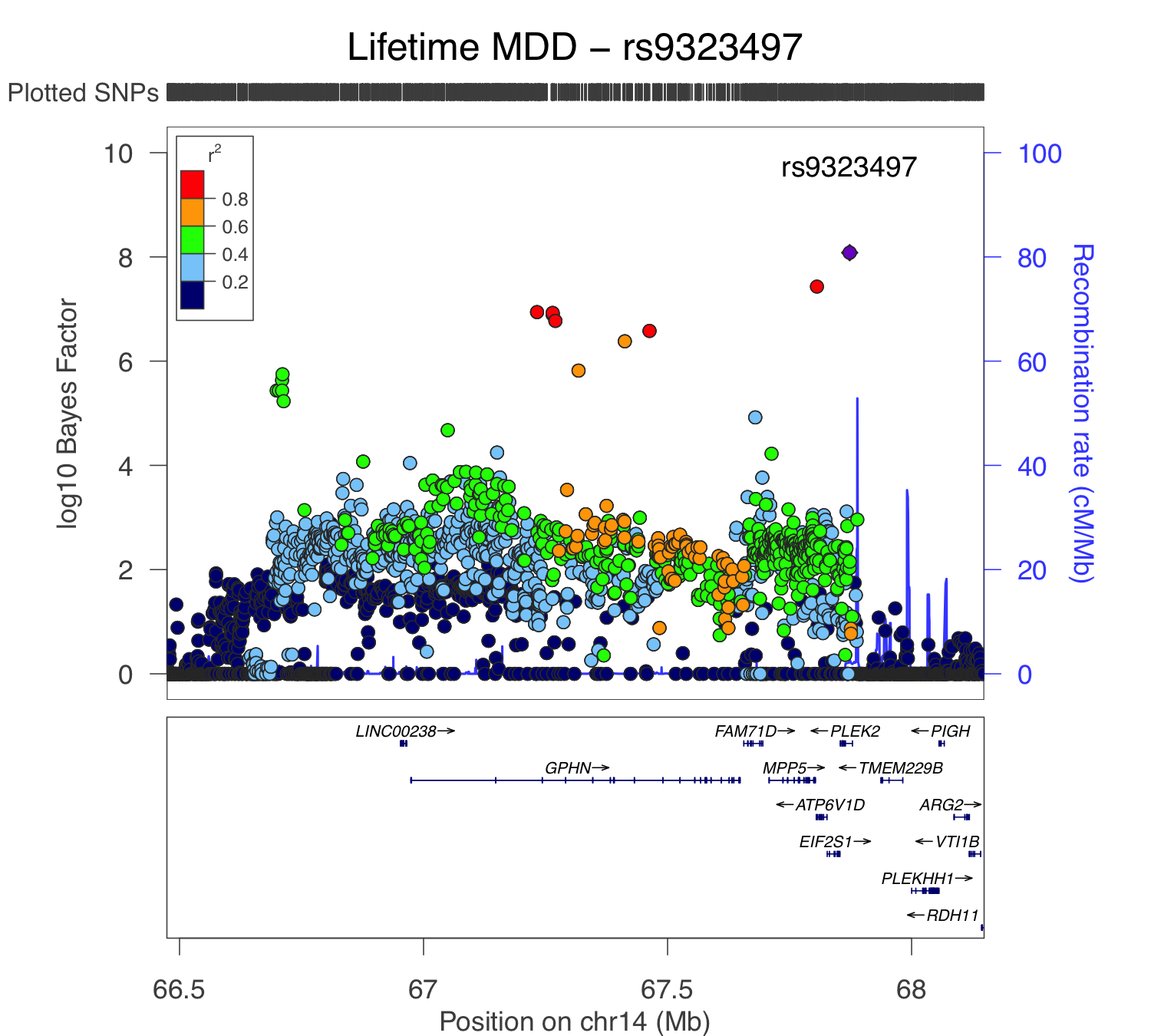

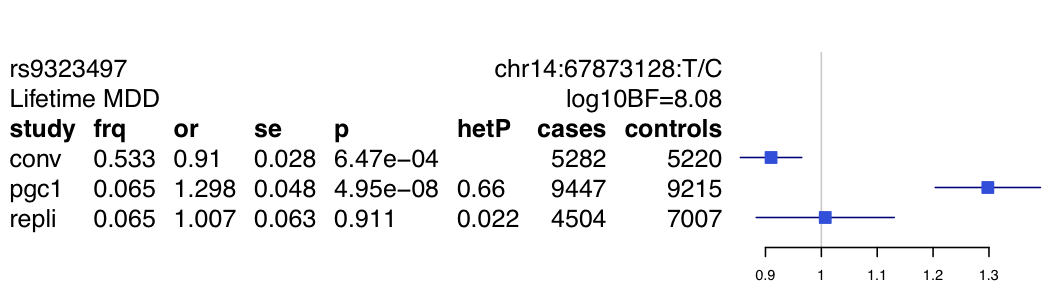


## *Supplemental Figure S5. Regional Association and Forest Plots for Females-only MDD***.** Panels and abbreviations are as described for Supplemental Figure 4.
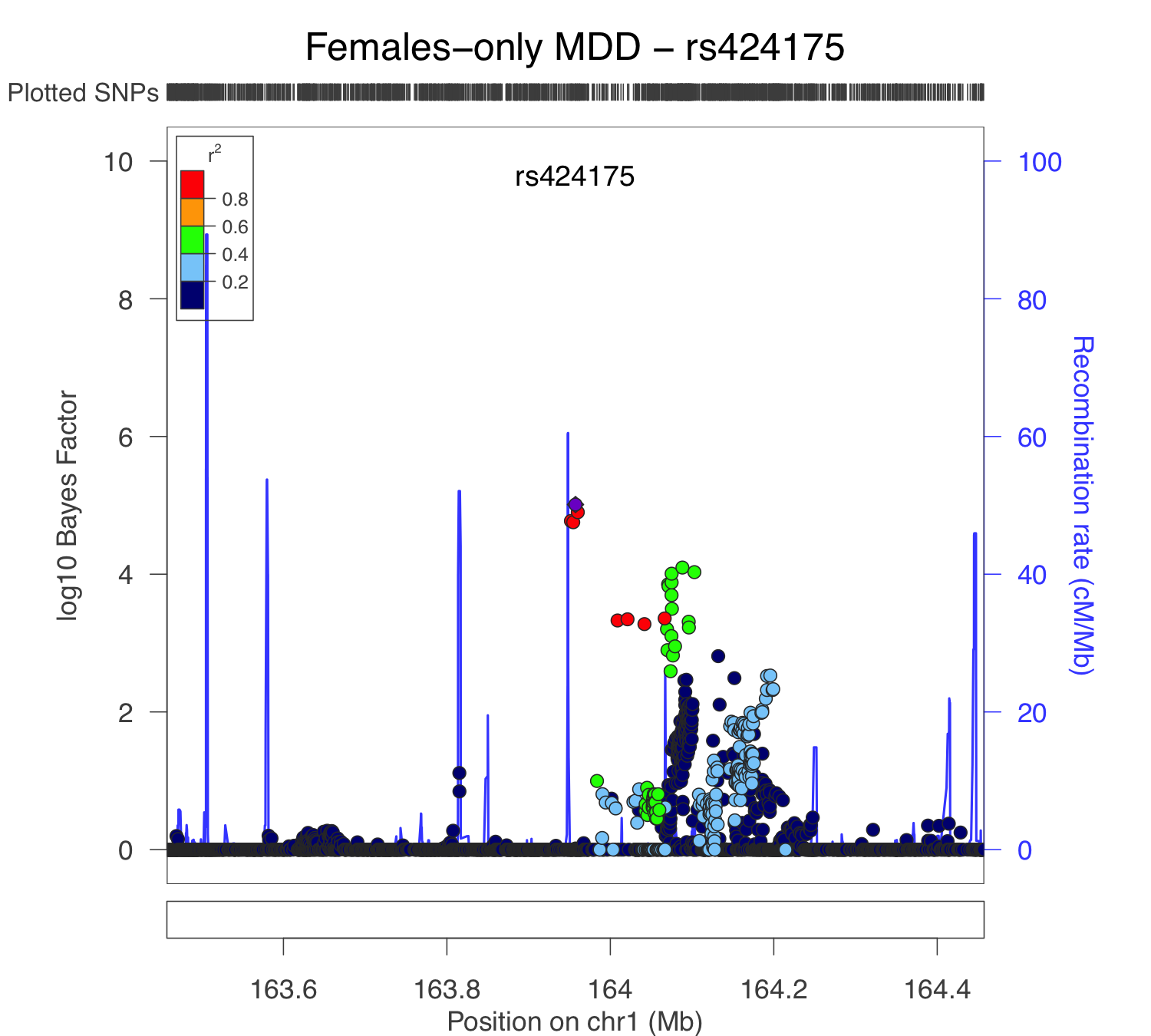

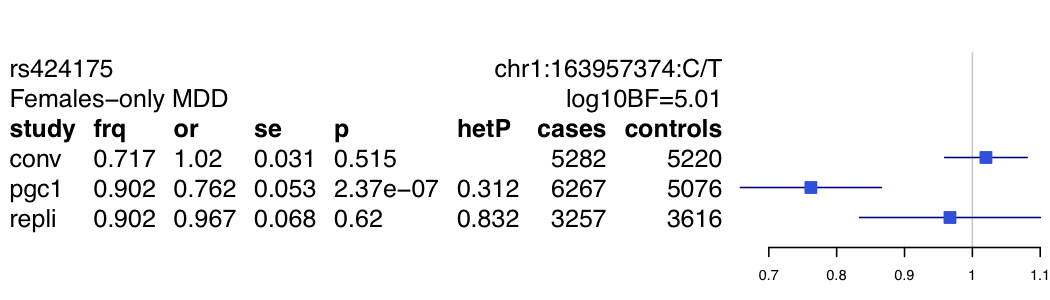

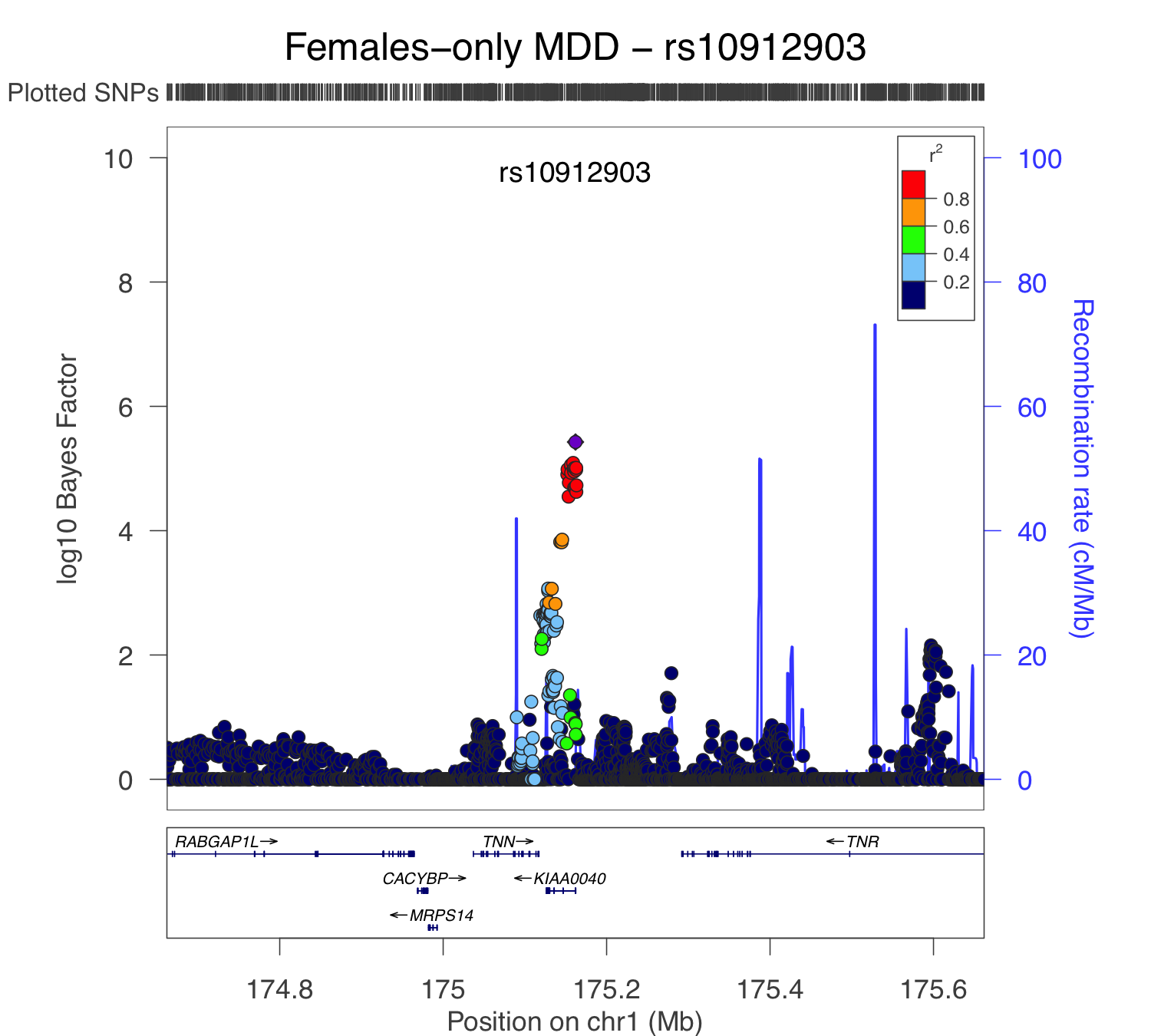

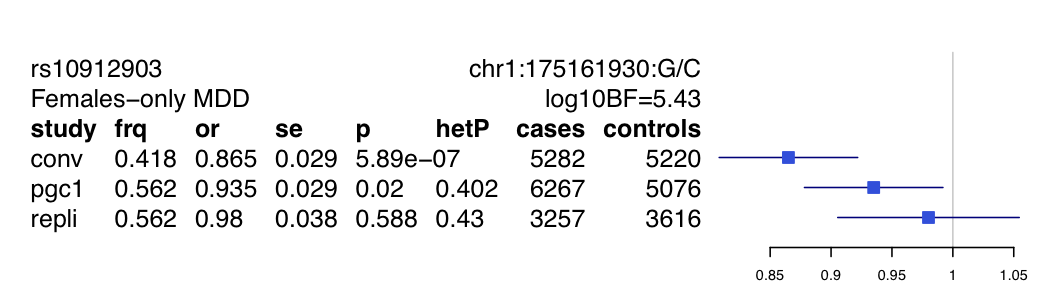

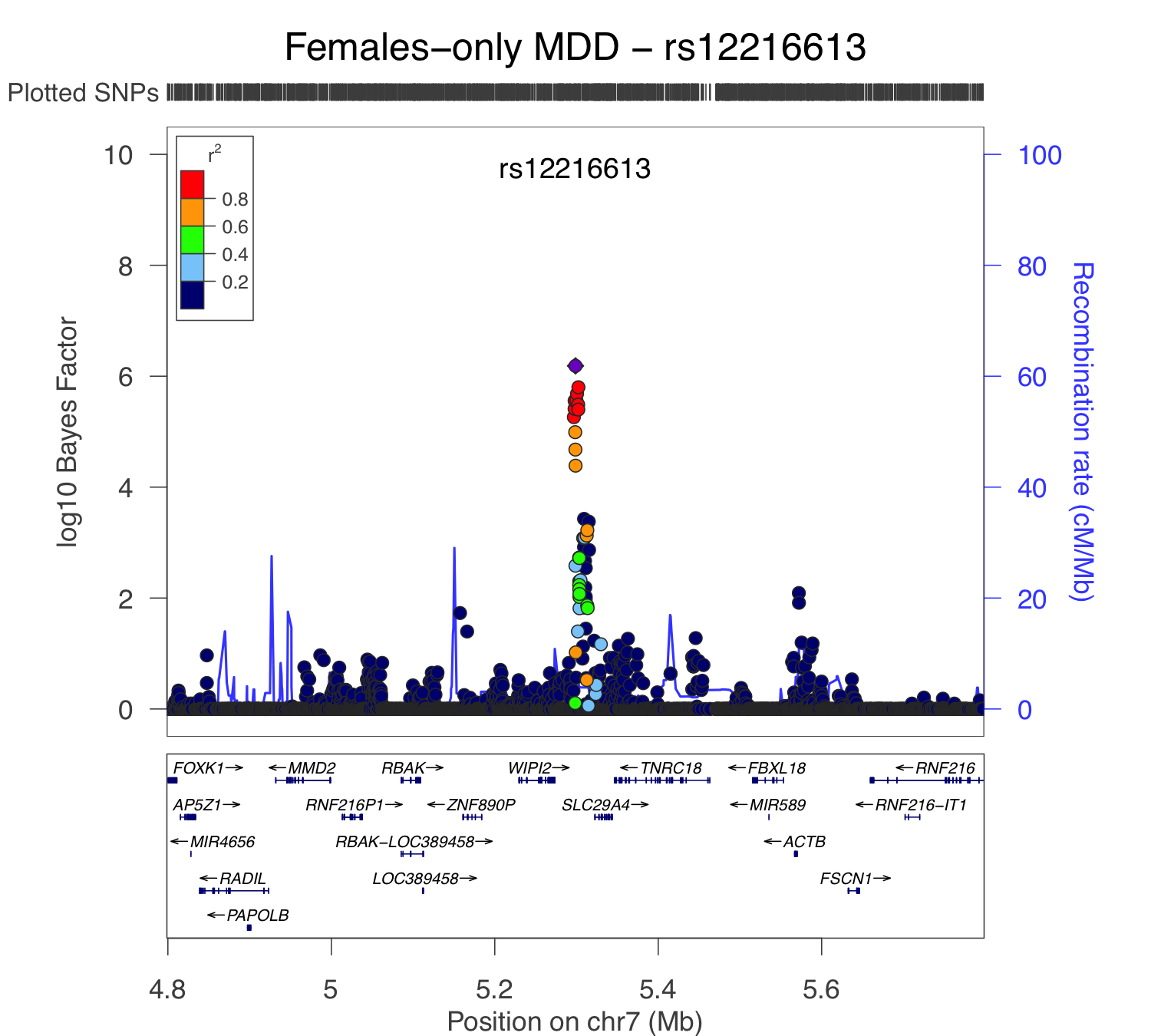

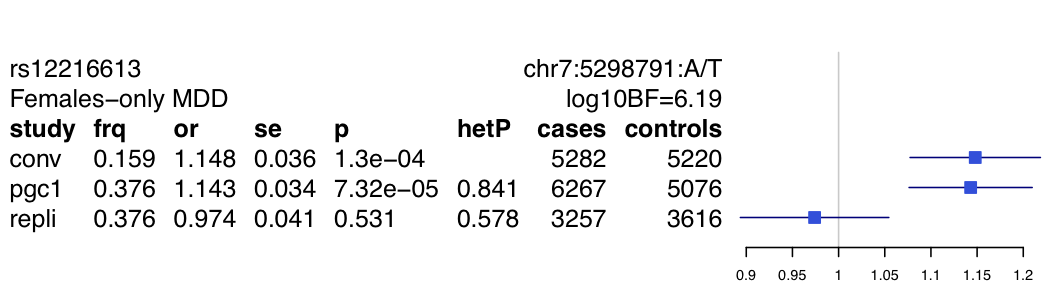

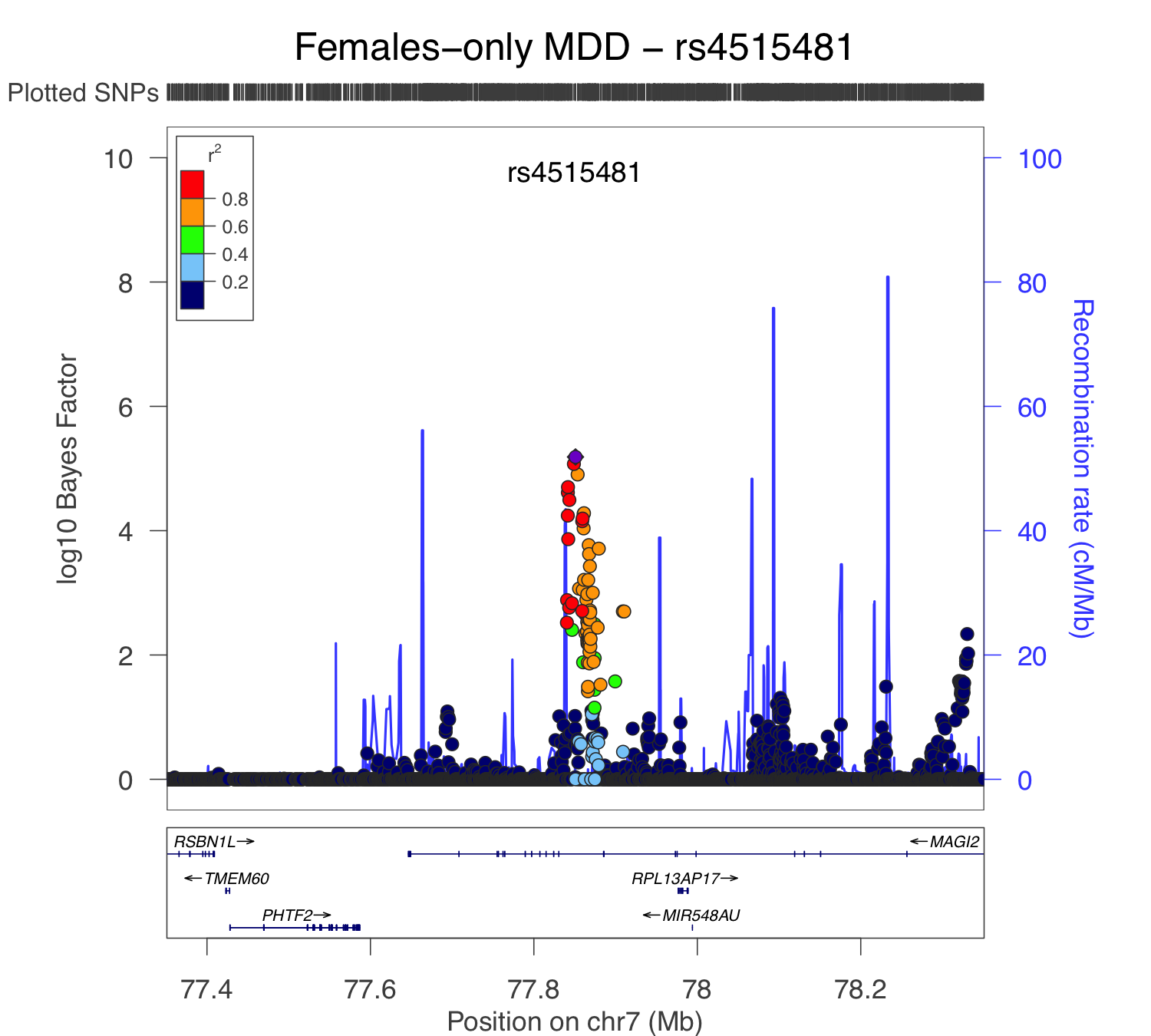

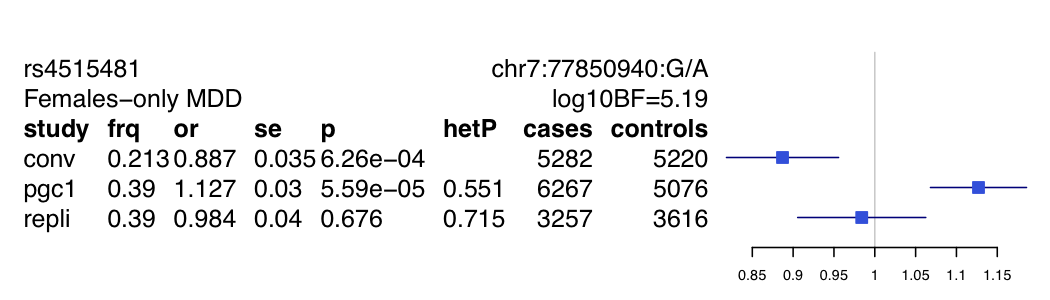

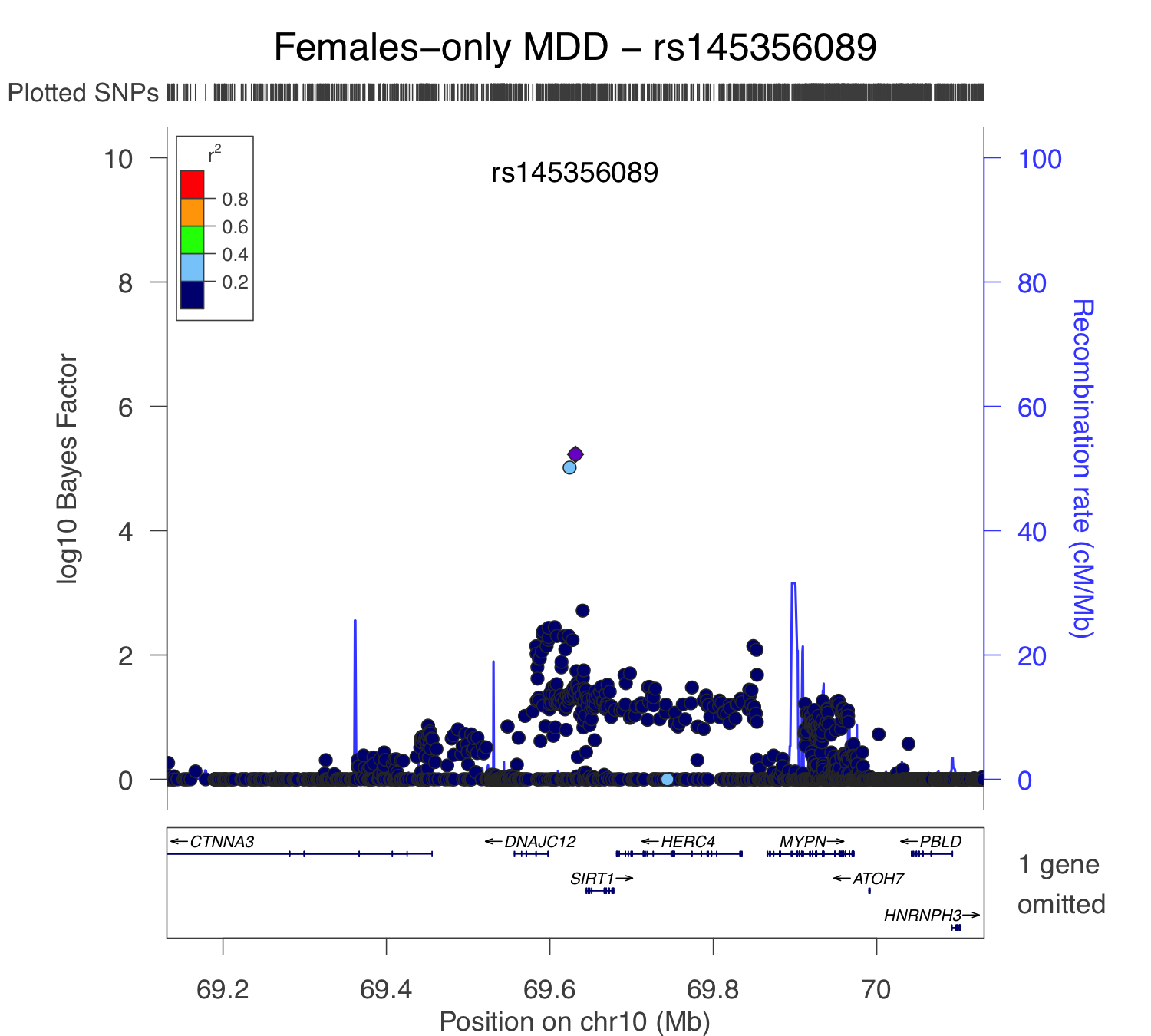

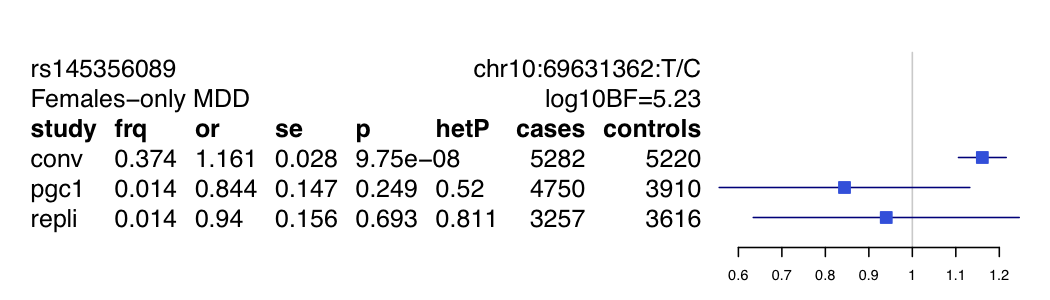

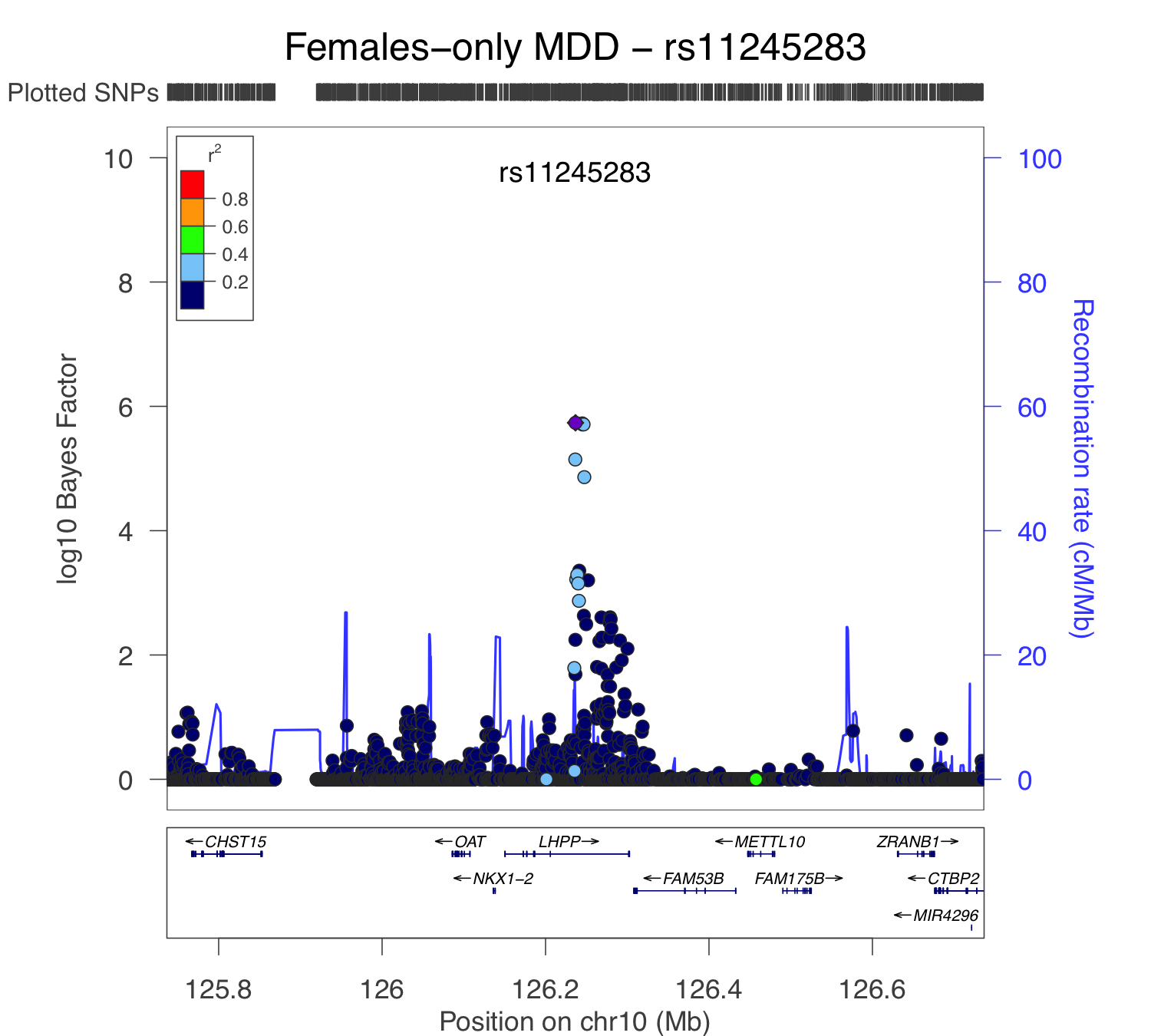

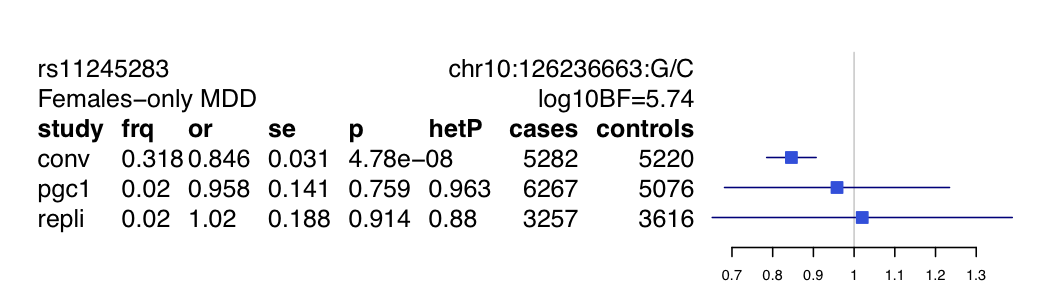

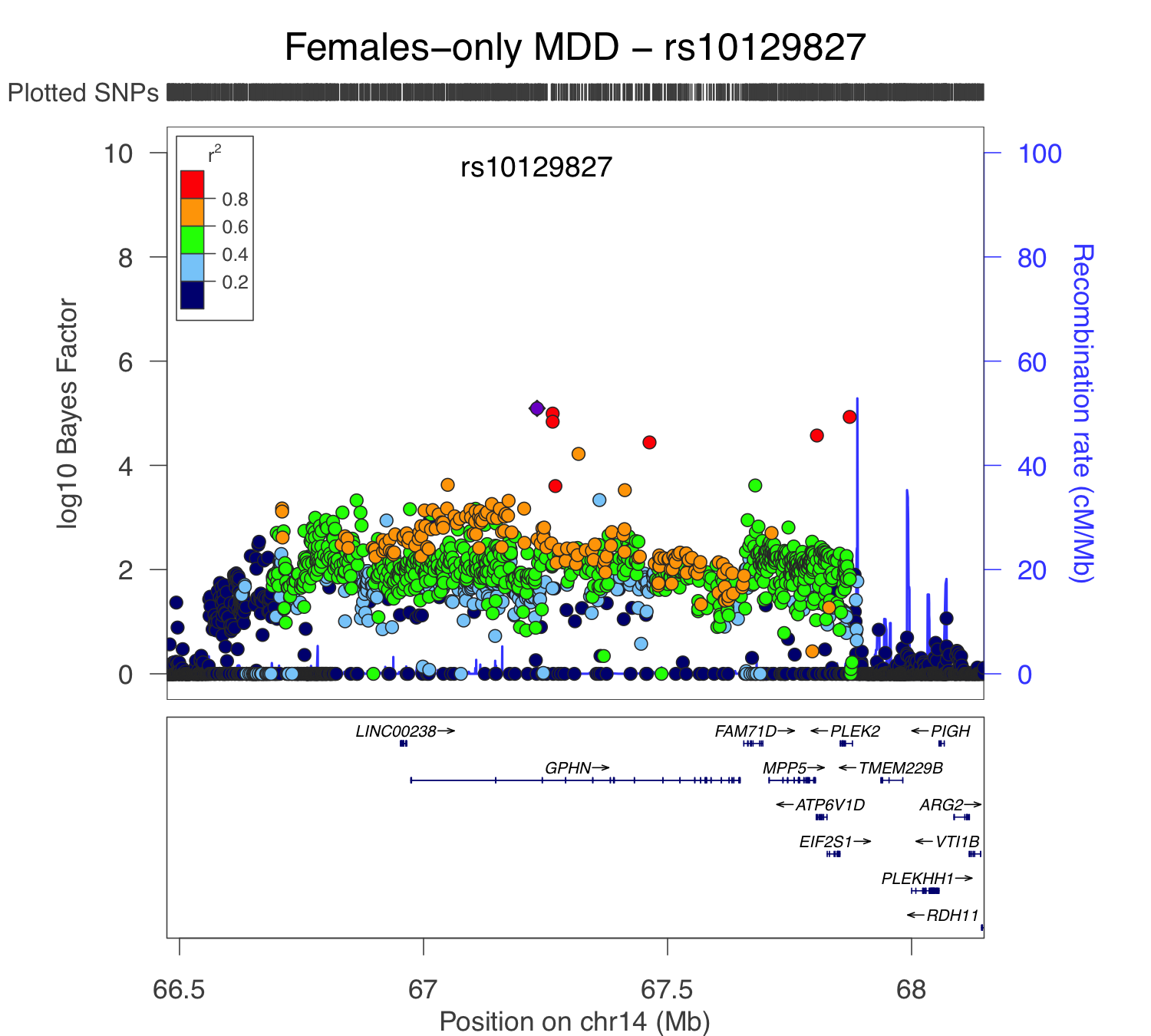

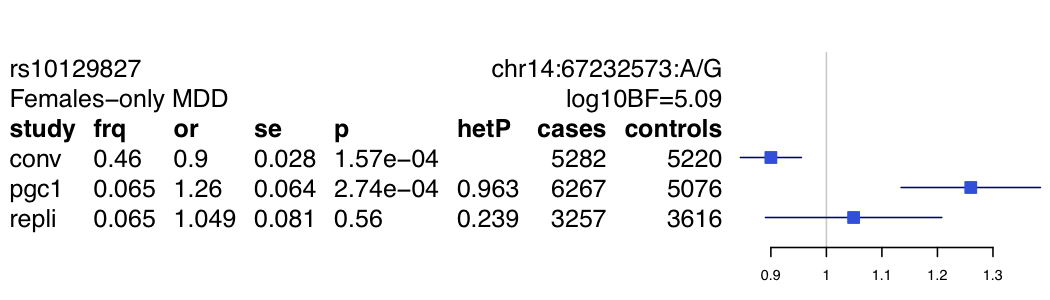


## *Supplemental Figure S6. Regional Association and Forest Plots for Recurrent MDD***.** Panels and abbreviations are as described for Supplemental Figures 4.
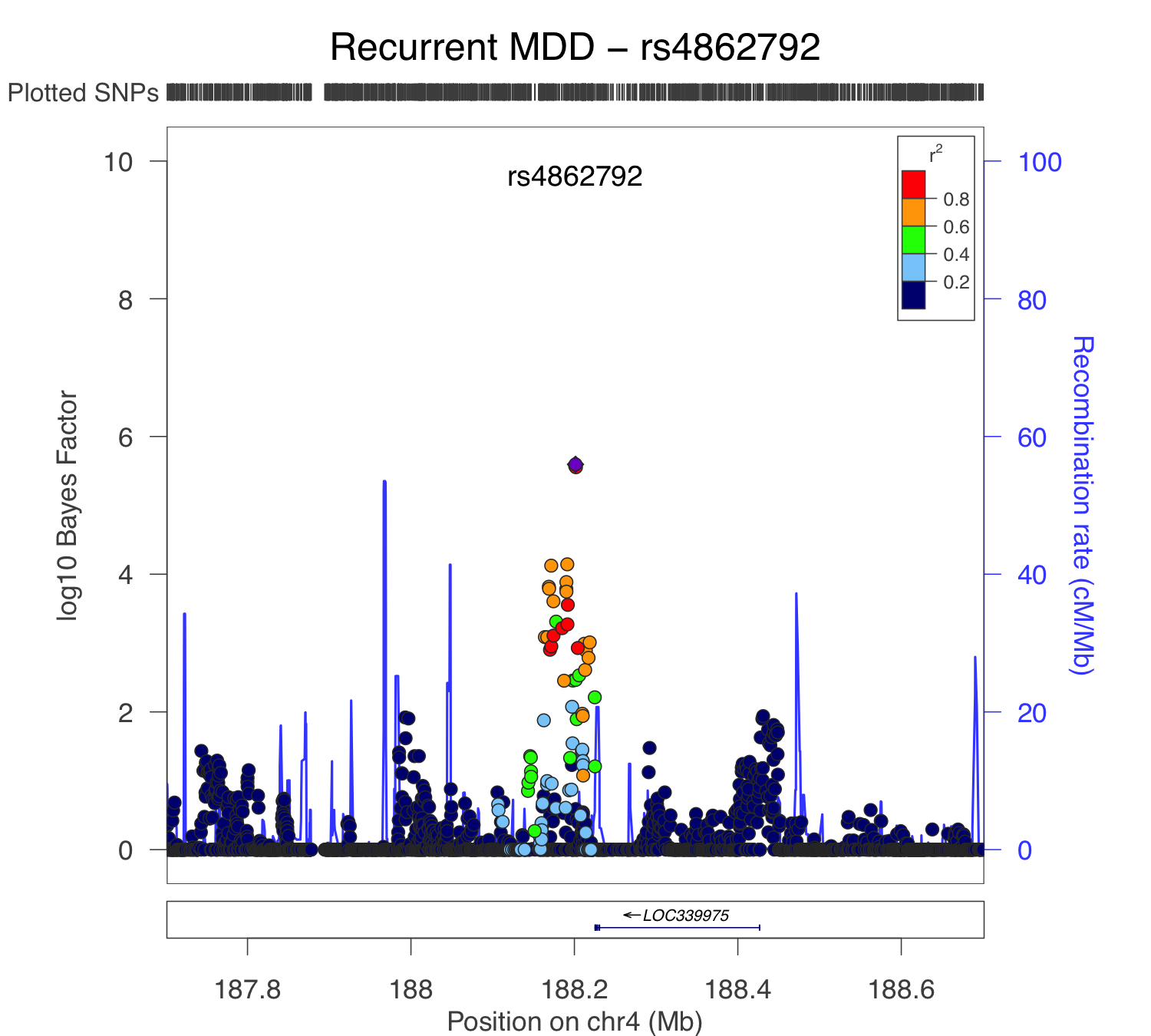

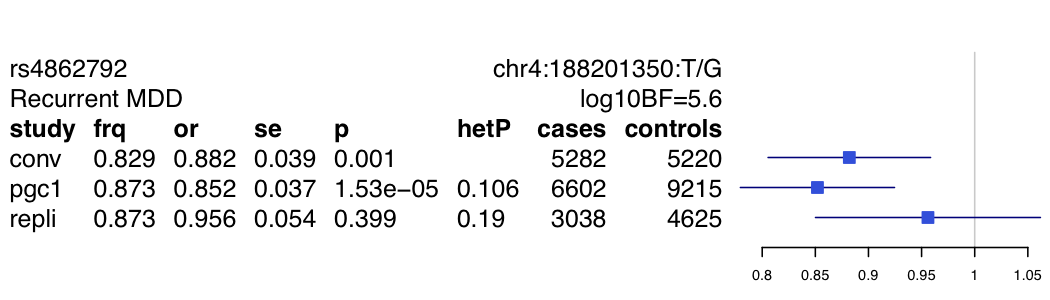

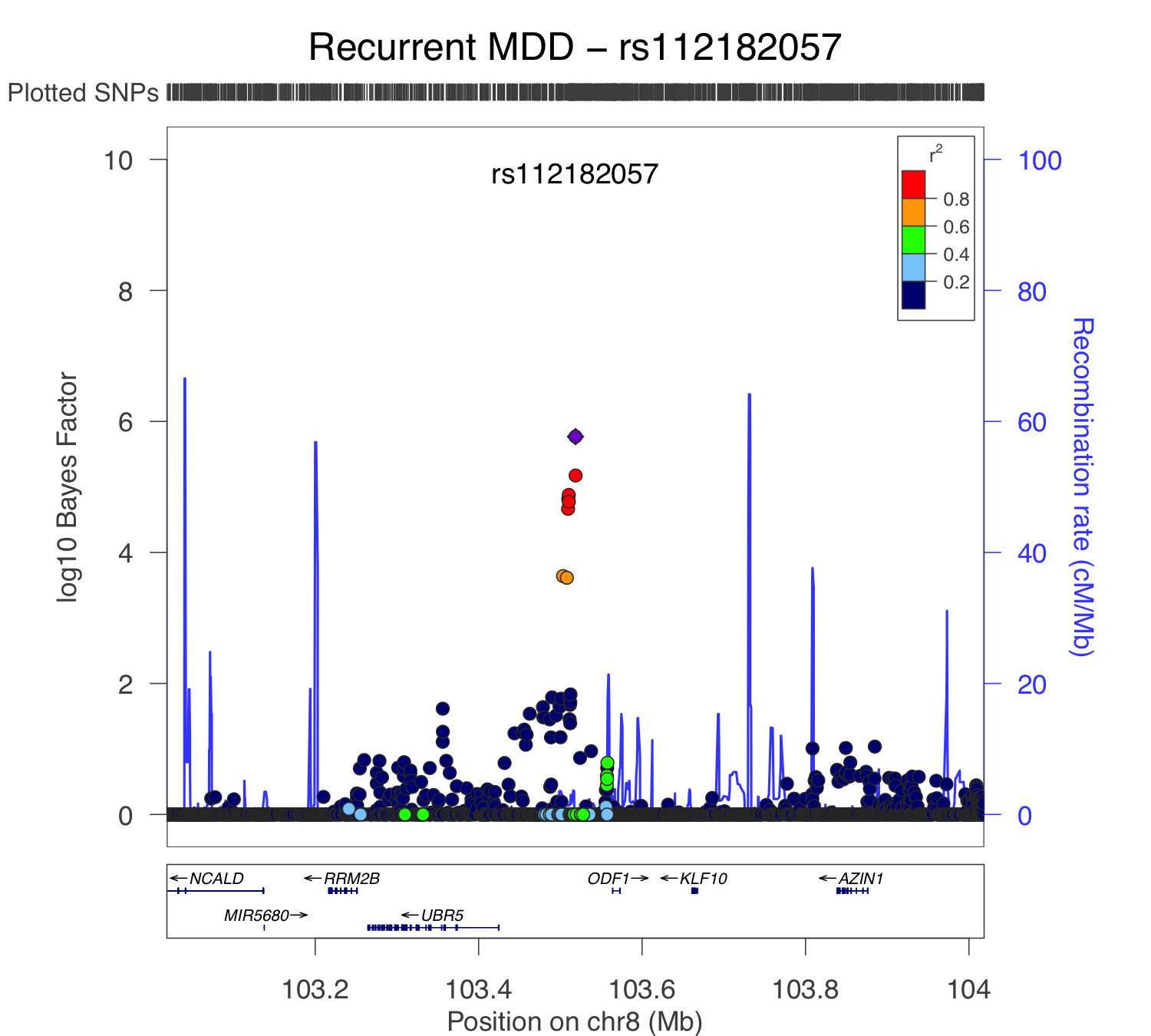

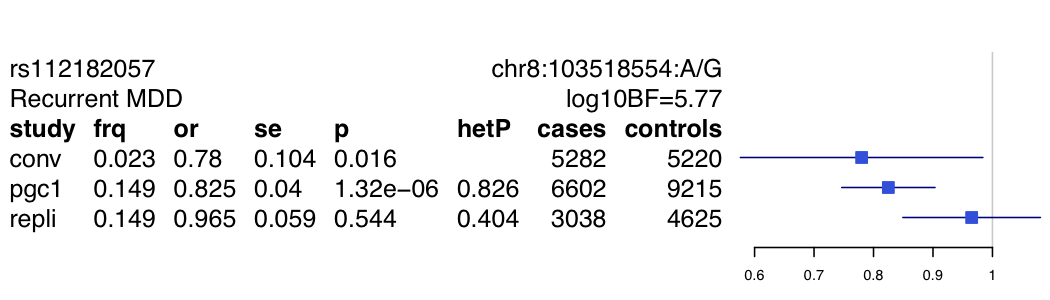

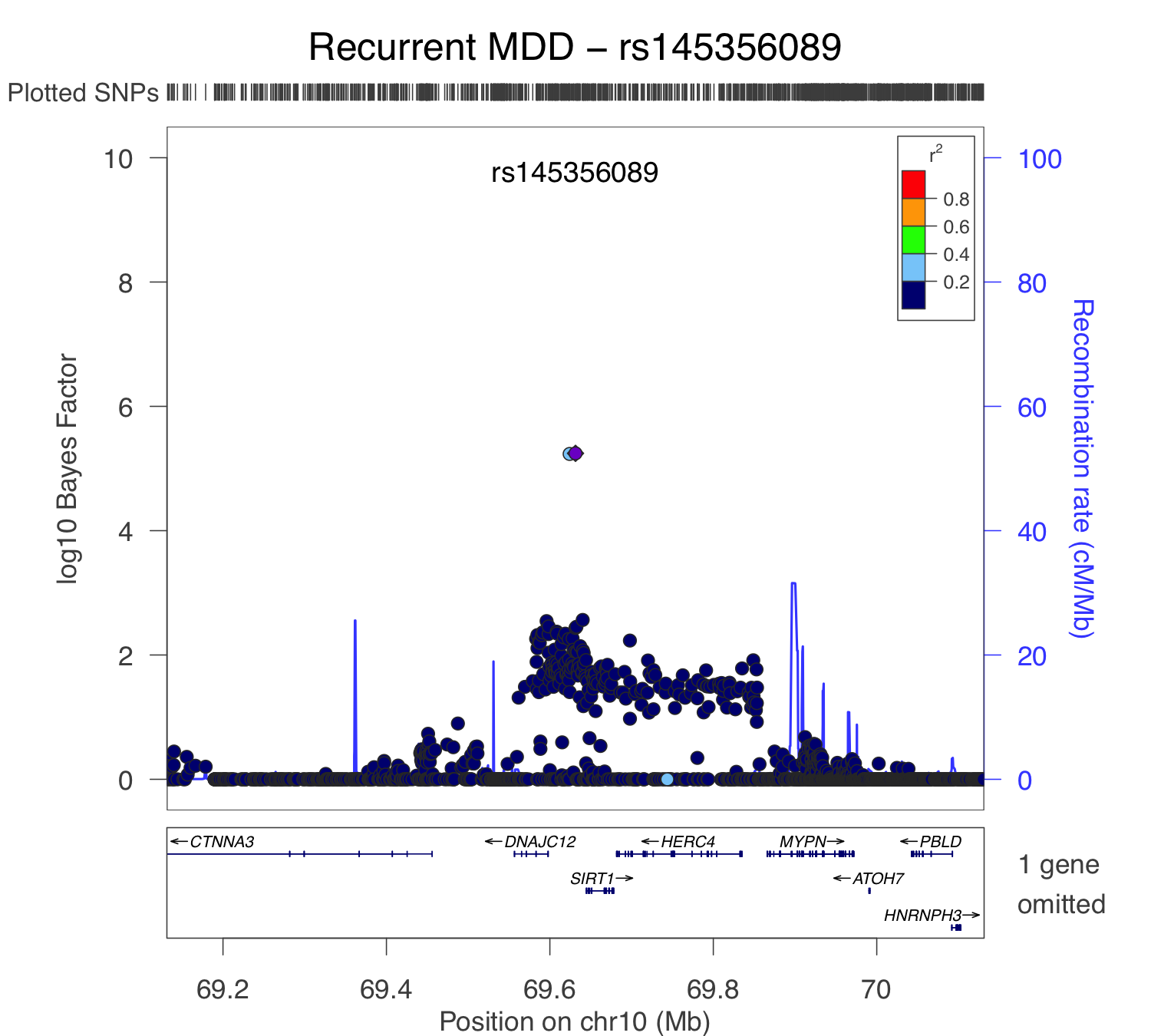

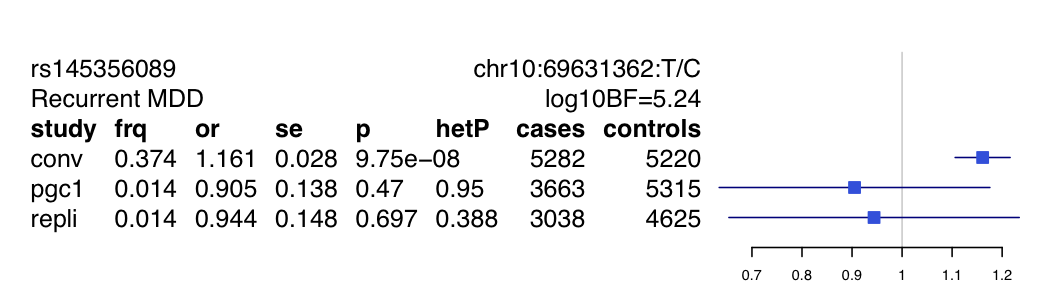

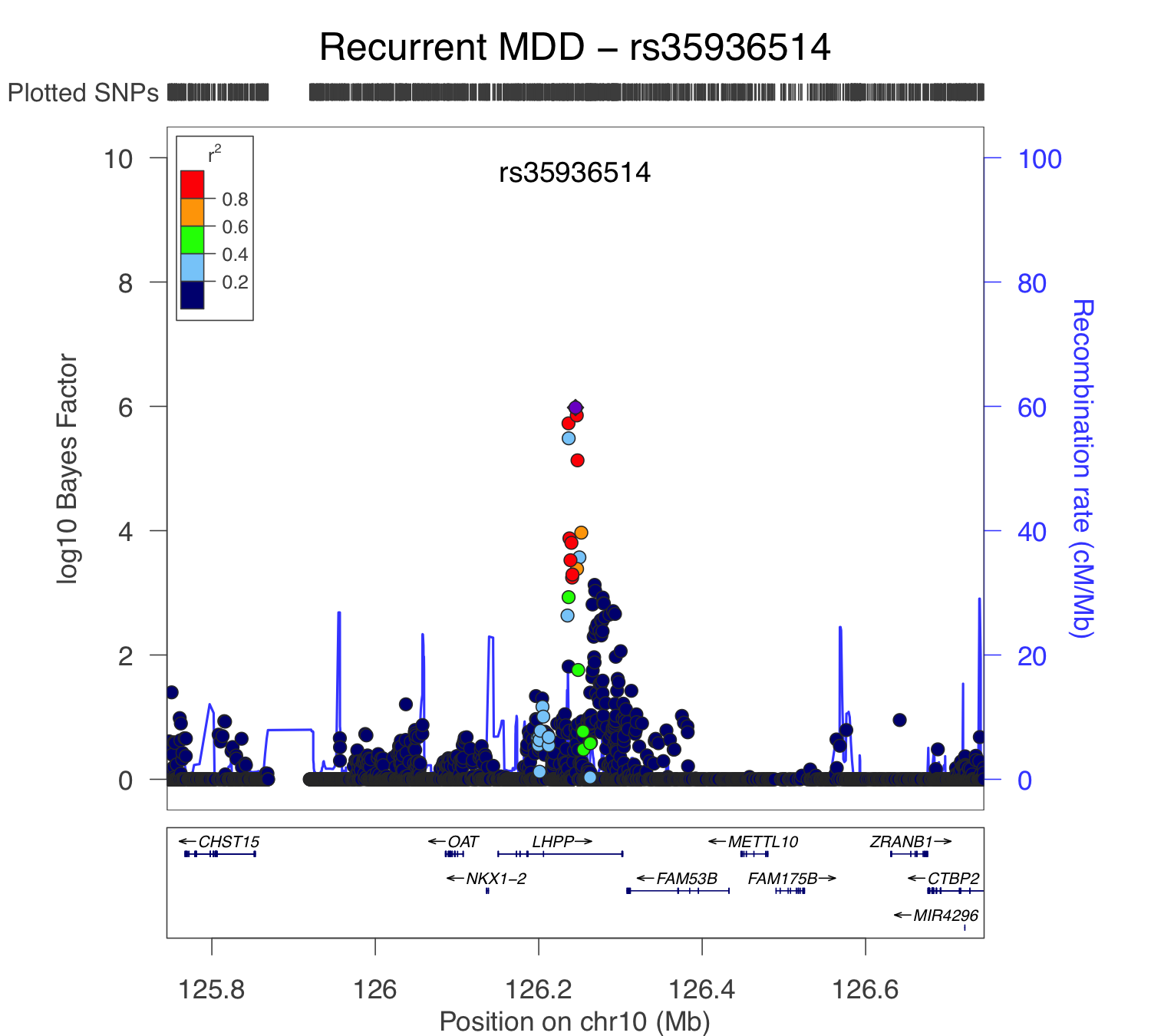

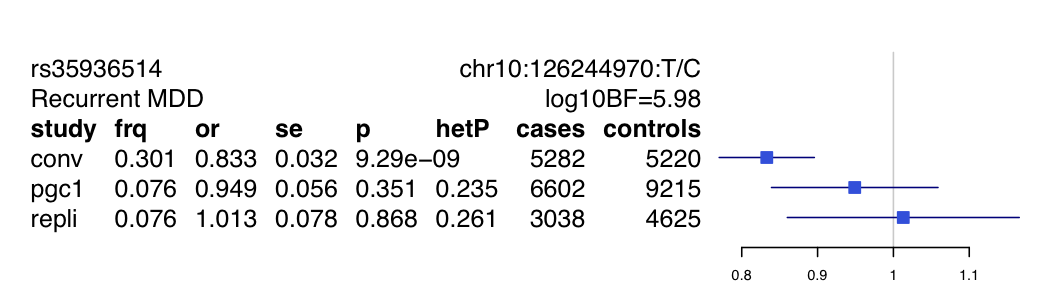

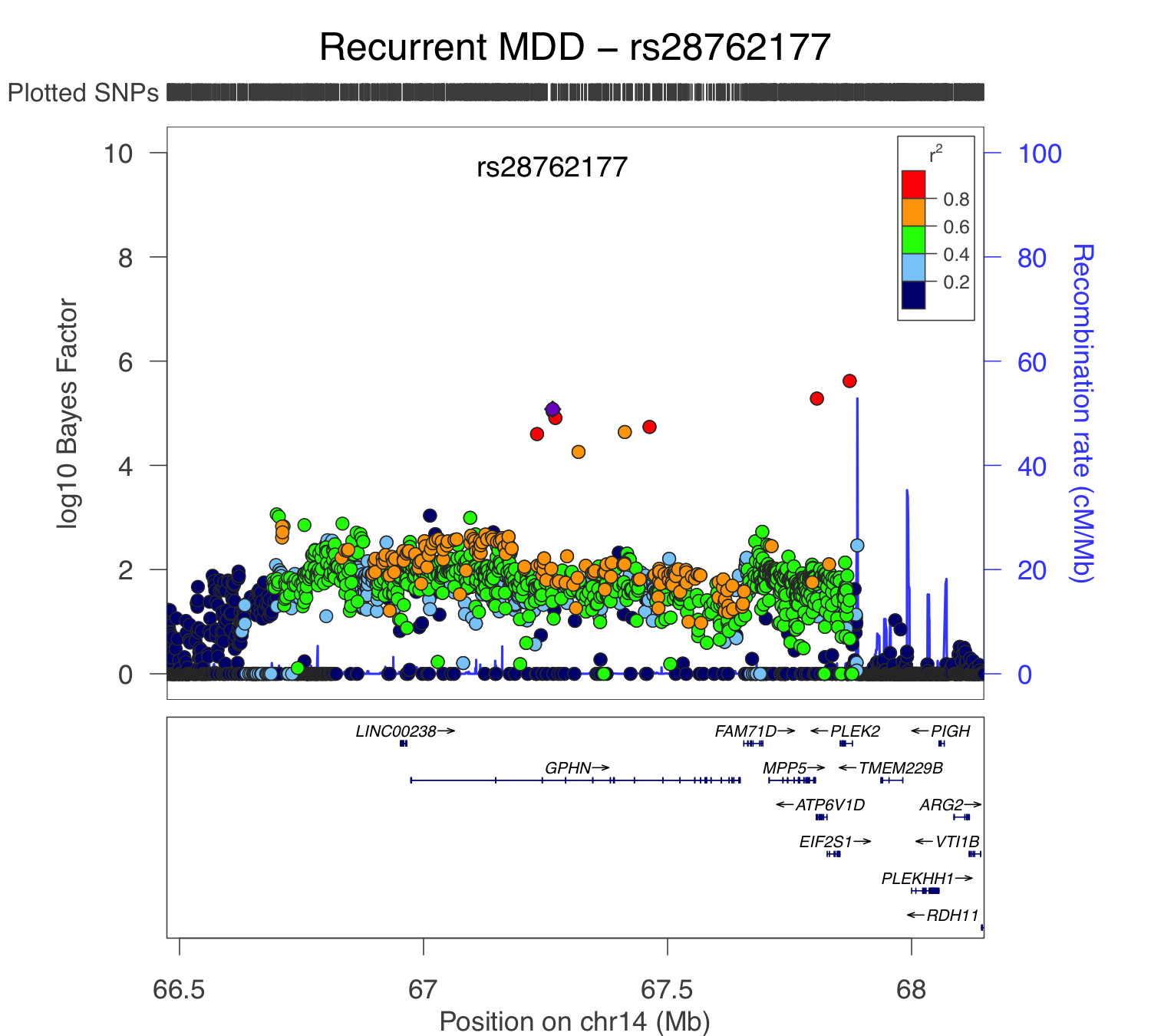

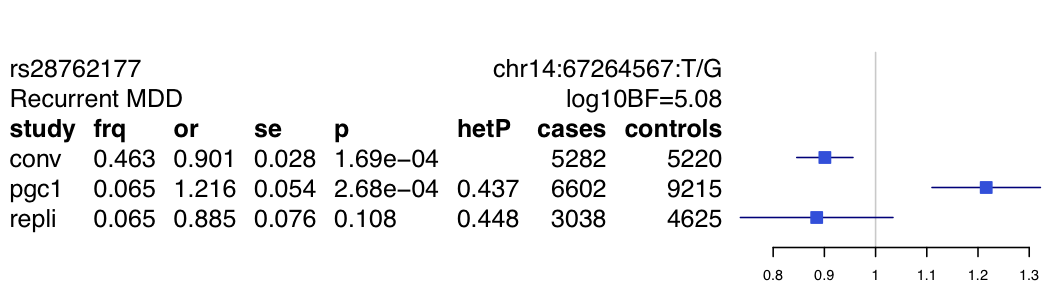

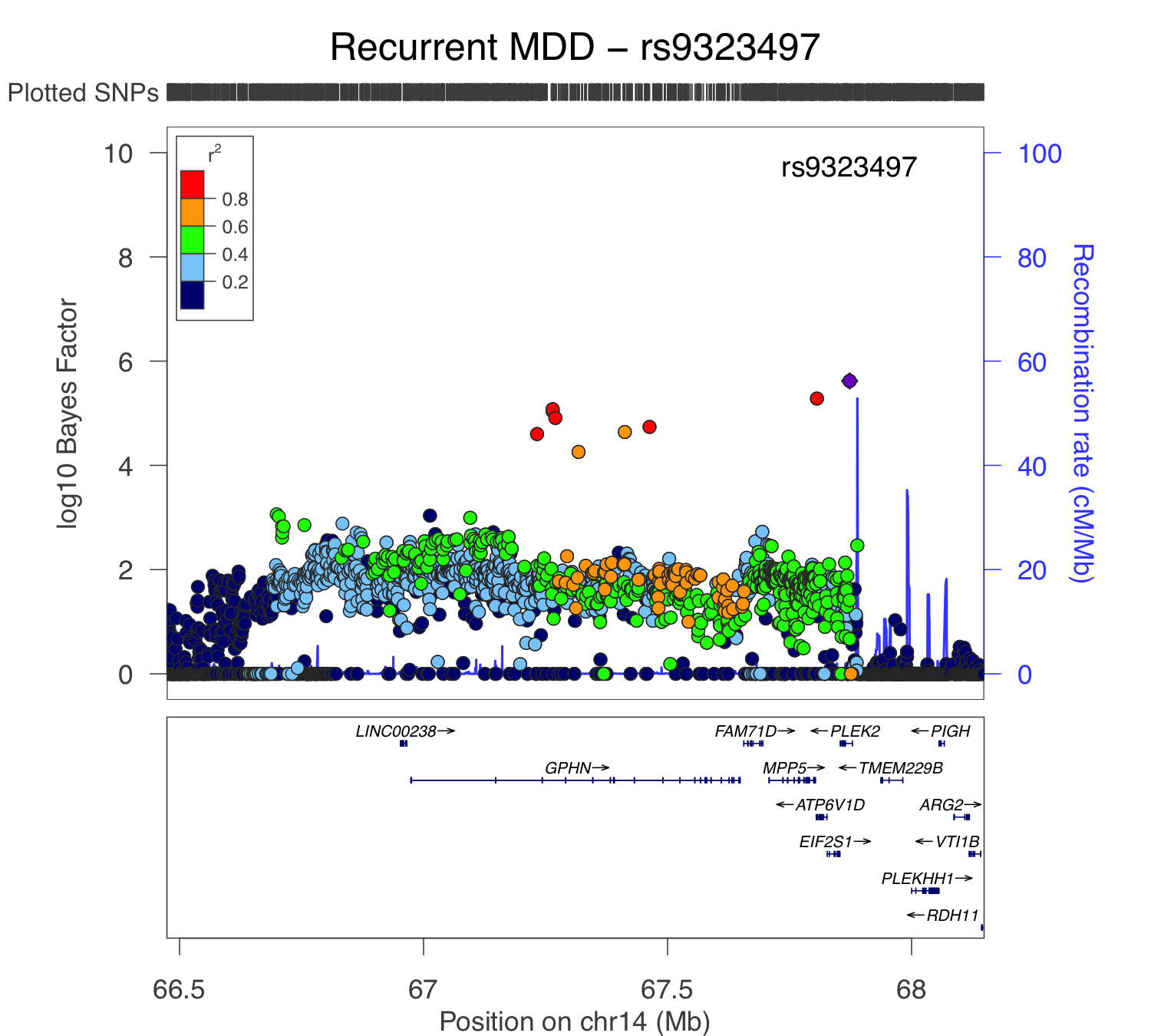

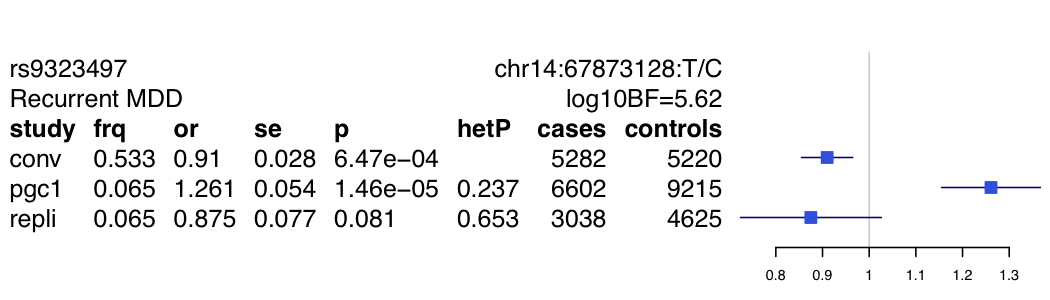

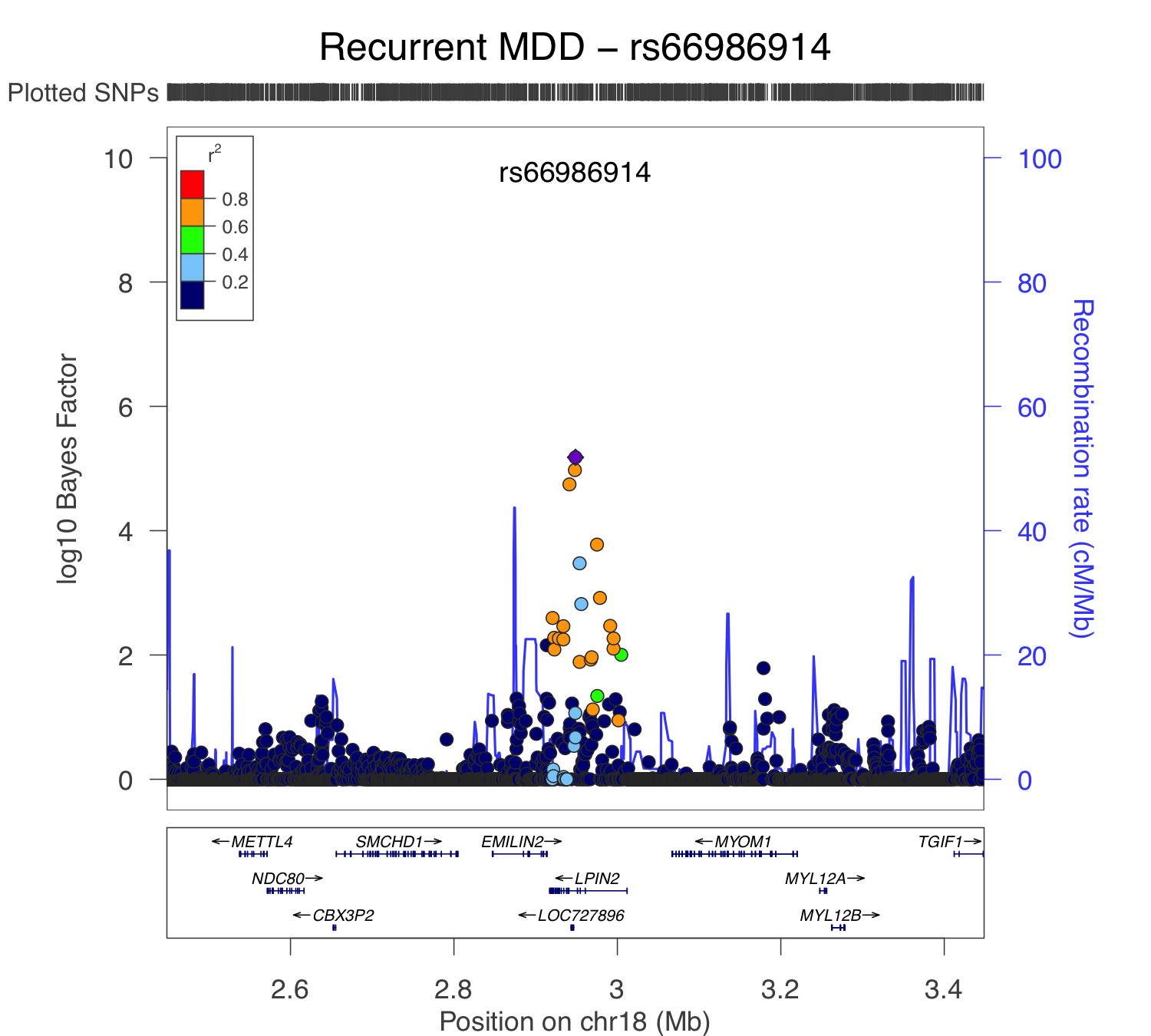

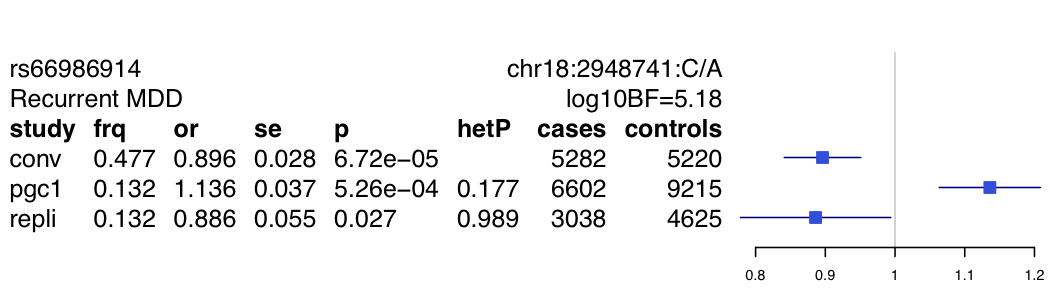


# **Supplemental Tables**

## *Supplemental Table S1. Variance in disease risk explained by PRS.* For PGC- and CONVERGE-trained polygenic scores based on varying P-value thresholds (*P_T_*), estimates of variance explained, in terms of Nagelkerke's pseudo-R-squared, are displayed.

| *P_T_* | PGC-trained | | | CONVERGE-trained | | |
| --- | --- | --- | --- | --- | --- | --- |
|  | MDD | Females-only | Recurrent | MDD | Females-only | Recurrent |
| 10^-5^ | 6.49×10^-6^ | 2.59×10^-5^ | 8.01×10^-5^ | 6.08×10^-4^ | 1.07×10^-3^ | 9.13×10^-4^ |
| 10^-4^ | 2.01×10^-4^ | 2.35×10^-4^ | 1.37×10^-5^ | 3.38×10^-4^ | 8.56×10^-4^ | 5.99×10^-4^ |
| 10^-3^ | 3.38×10^-4^ | 4.24×10^-4^ | 1.38×10^-3^ | 6.83×10^-6^ | 2.16×10^-4^ | 1.81×10^-4^ |
| 10^-2^ | 7.46×10^-4^ | 7.64×10^-5^ | 1.83×10^-3^ | 1.12×10^-4^ | 6.65×10^-4^ | 2.63×10^-4^ |
| 0.1 | 5.83×10^-4^ | 3.01×10^-4^ | 1.93×10^-3^ | 3.18×10^-4^ | 1.11×10^-3^ | 4.67×10^-4^ |
| 0.2 | 6.29×10^-4^ | 5.52×10^-4^ | 2.01×10^-3^ | 2.57×10^-4^ | 7.82×10^-4^ | 3.35×10^-4^ |
| 0.3 | 6.92×10^-4^ | 6.44×10^-4^ | 1.88×10^-3^ | 2.12×10^-4^ | 7.07×10^-4^ | 3.04×10^-4^ |
| 0.4 | 5.43×10^-4^ | 6.73×10^-4^ | 1.76×10^-3^ | 1.63×10^-4^ | 6.04×10^-4^ | 2.79×10^-4^ |
| 0.5 | 4.53×10^-4^ | 7.12×10^-4^ | 1.78×10^-3^ | 1.97×10^-4^ | 7.16×10^-4^ | 3.38×10^-4^ |

## *Supplemental Table S2. Significance of association between PRS and affection status.* For PGC- and CONVERGE-trained polygenic scores based on varying *P*-value thresholds (*P_T_*), significance estimates (*i.e.,* *P*-values) as assessed by logistic regression are displayed.

| *P_T_* | PGC-trained | | | CONVERGE-trained | | |
| --- | --- | --- | --- | --- | --- | --- |
|  | MDD | Females-only | Recurrent | MDD | Females-only | Recurrent |
| 10^-5^ | 0.821 | 0.651 | 0.426 | 0.007 | 0.005 | 0.002 |
| 10^-4^ | 0.207 | 0.172 | 0.742 | 0.043 | 0.012 | 0.012 |
| 10^-3^ | 0.102 | 0.067 | 9.57×10^-^*^4^ | 0.773 | 0.205 | 0.169 |
| 10^-2^ | 0.015 | 0.437 | 1.42×10^-4^* | 0.243 | 0.026 | 0.098 |
| 0.1 | 0.032 | 0.122 | 9.36×10^-5^* | 0.050 | 0.004 | 0.027 |
| 0.2 | 0.026 | 0.037 | 6.56×10^-5^* | 0.077 | 0.016 | 0.061 |
| 0.3 | 0.019 | 0.024 | 1.13×10^-4^* | 0.109 | 0.022 | 0.075 |
| 0.4 | 0.038 | 0.021 | 1.94×10^-4^* | 0.160 | 0.034 | 0.087 |
| 0.5 | 0.058 | 0.018 | 1.76×10^-4^* | 0.123 | 0.021 | 0.060 |

## **indicates a significant comparison after Bonferroni correction for 27 tests.*

*Supplemental Table S3. Regression coefficients for PRS.* For PGC- and CONVERGE-trained scores based on varying *P*-value thresholds (*P_T_*), beta estimates (SE) from logistic regression are displayed.

| *P_T_* | PGC-trained | | | CONVERGE-trained | | |
| --- | --- | --- | --- | --- | --- | --- |
|  | MDD | Females-only | Recurrent | MDD | Females-only | Recurrent |
| 10^-5^ | 0.004 (0.019) | 0.085 (0.035) | 0.007 (0.065) | 0.087 (0.032) | 0.117 (0.041) | 0.111 (0.036) |
| 10^-4^ | 0.012 (0.010) | 0.008 (0.018) | 0.017 (0.021) | 0.027 (0.013) | 0.044 (0.017) | 0.038 (0.015) |
| 10^-3^ | 0.007 (0.004) | 0.010 (0.008) | 0.003 (0.009) | 0.002 (0.006) | 0.010 (0.008) | 0.009 (0.007) |
| 10^-2^ | 0.007 (0.003) | 0.006 (0.003) | 0.013 (0.004) | 0.004 (0.004) | 0.010 (0.005) | 0.007 (0.004) |
| 0.1 | 0.005 (0.002) | 0.002 (0.002) | 0.010 (0.003) | 0.006 (0.003) | 0.011 (0.004) | 0.008 (0.003) |
| 0.2 | 0.005 (0.002) | 0.003 (0.002) | 0.008 (0.002) | 0.005 (0.003) | 0.009 (0.004) | 0.006 (0.003) |
| 0.3 | 0.005 (0.002) | 0.003 (0.002) | 0.008 (0.002) | 0.004 (0.003) | 0.008 (0.003) | 0.005 (0.003) |
| 0.4 | 0.004 (0.002) | 0.004 (0.002) | 0.007 (0.002) | 0.004 (0.003) | 0.007 (0.003) | 0.005 (0.003) |
| 0.5 | 0.004 (0.002) | 0.004 (0.002) | 0.007 (0.002) | 0.004 (0.003) | 0.007 (0.003) | 0.005 (0.003) |

##

## *Supplemental Table S4.* *Binomial Sign Test.* For varying *P*-value thresholds (*P_T_*), the number of SNPs, *fraction* of these with consistent direction of effect, and significance of a one-sided binomial test (*P_binom_*).

| *P_T_* | | PGC-trained | | | CONVERGE-trained | | |
| --- | --- | --- | --- | --- | --- | --- | --- |
|  |  | MDD | Females-only | Recurrent | MDD | Females-only | Recurrent |
| 10^-5^ | SNPs | 10 | 16 | 10 | 18 | 18 | 18 |
|  | fraction | 0.6000 | 0.6875 | 0.7000 | 0.6111 | 0.5556 | 0.6667 |
|  | *P_binom_* | 0.377 | 0.105 | 0.172 | 0.240 | 0.407 | 0.119 |
| 10^-4^ | SNPs | 111 | 104 | 129 | 101 | 101 | 101 |
|  | fraction | 0.5045 | 0.5577 | 0.4806 | 0.5842 | 0.6139 | 0.5842 |
|  | *P_binom_* | 0.500 | 0.140 | 0.701 | 0.0555 | 0.0140 | 0.0555 |
| 10^-3^ | SNPs | 756 | 835 | 900 | 769 | 768 | 770 |
|  | fraction | 0.5000 | 0.5473 | 0.4967 | 0.5189 | 0.5378 | 0.5286 |
|  | *P_binom_* | 0.515 | 3.46×10^-3^ | 0.592 | 0.156 | 0.0198 | 0.0606 |
| 10^-2^ | SNPs | 5226 | 5980 | 6210 | 5433 | 5441 | 5447 |
|  | fraction | 0.5138 | 0.5162 | 0.5161 | 0.5041 | 0.5082 | 0.5095 |
|  | *P_binom_* | 0.0240 | 6.28×10^-3^ | 5.78×10^-3^ | 0.275 | 0.116 | 0.0835 |
| 0.1 | SNPs | 29737 | 33747 | 34449 | 31921 | 31984 | 32052 |
|  | fraction | 0.5070 | 0.5045 | 0.5108 | 0.5058 | 0.5086 | 0.5058 |
|  | *P_binom_* | 8.18×10^-3^ | 0.0490 | 3.05×10^-5^* | 0.0197 | 1.11×10^-3^* | 0.0186 |
| 0.2 | SNPs | 46743 | 52681 | 53087 | 50427 | 50608 | 50665 |
|  | fraction | 0.5072 | 0.5042 | 0.5081 | 0.5048 | 0.5066 | 0.5040 |
|  | *P_binom_* | 1.00×10^-3^* | 0.0276 | 1.05×10^-4^* | 0.0156 | 1.51×10^-3^* | 0.0363 |
| 0.3 | SNPs | 59395 | 65963 | 66292 | 64371 | 64581 | 64678 |
|  | fraction | 0.5046 | 0.5049 | 0.5069 | 0.5027 | 0.5051 | 0.5030 |
|  | *P_binom_* | 0.0123 | 6.08×10^-3^ | 1.96×10^-4^* | 0.0863 | 5.15×10^-3^ | 0.0650 |
| 0.4 | SNPs | 69461 | 76339 | 76439 | 75451 | 75689 | 75843 |
|  | fraction | 0.5028 | 0.5044 | 0.5055 | 0.5016 | 0.5039 | 0.5028 |
|  | *P_binom_* | 0.0695 | 7.50×10^-3^ | 1.25×10^-3^* | 0.189 | 0.0169 | 0.0609 |
| 0.5 | SNPs | 77445 | 84257 | 84393 | 84277 | 84603 | 84745 |
|  | fraction | 0.5010 | 0.5047 | 0.5046 | 0.5028 | 0.5042 | 0.5035 |
|  | *P_binom_* | 0.283 | 3.46×10^-3^ | 4.10×10^-3^ | 0.0505 | 7.32×10^-3^ | 0.0196 |

## **indicates a significant comparison after Bonferroni correction for 27 tests.*

## *Supplemental Table S5.* *Trans-ancestry meta-analysis results for lifetime MDD.* For each SNP, *Polymorphism* gives the chromosome, genomic coordinates (GRCh37), and alleles; effects are with respect to the first listed allele, and overall significance is given in terms of the log Bayes Factor in favor of association. For CONVERGE and PGC, *Freq* is the frequency of the tested allele in East-Asian and European subjects from the 1000 Genomes Project, respectively.

| SNP | Polymorphism | Discovery Phase | | | | | | | Replication Phase | |
| --- | --- | --- | --- | --- | --- | --- | --- | --- | --- | --- |
|  |  | CONVERGE | | | PGC | | | log_10_BF |  |  |
|  |  | Freq | OR (SE) | *P* | Freq | OR (SE) | *P* |  | OR (SE) | *P* |
| rs13088706 | 3:72665004:G/C | 0.063 | 0.846 (0.058) | 3.84×10^-3^ | 0.234 | 0.901 (0.024) | 1.64×10^-5^ | 5.086 | 1.026 (0.033) | 0.440 |
| rs7647854 | 3:184876783:A/G | 0.785 | 0.925 (0.033) | 0.017 | 0.815 | 0.860 (0.030) | 6.32×10^-7^ | 5.609 | 0.961 (0.040) | 0.317 |
| rs4515481 | 7:77850940:G/A | 0.213 | 0.887 (0.035) | 6.26×10^-4^ | 0.391 | 1.097 (0.023) | 4.64×10^-5^ | 5.166 | 0.982 (0.031) | 0.552 |
| rs56197202 | 8:23459043:T/C | 0.254 | 0.857 (0.032) | 1.02×10^-6^ | 0.310 | 0.936 (0.024) | 5.63×10^-3^ | 5.460 | 0.975 (0.032) | 0.436 |
| rs112182057 | 8:103518554:A/G | 0.023 | 0.780 (0.104) | 0.016 | 0.149 | 0.843 (0.035) | 1.17×10^-6^ | 5.739 | 0.995 (0.047) | 0.911 |
| rs12415800 | 10:69624180:A/G | 0.376 | 1.161 (0.028) | 8.90×10^-8^ | 0.026 | 0.996 (0.090) | 0.965 | 5.099 | 0.910 (0.125) | 0.451 |
| rs35936514 | 10:126244970:T/C | 0.301 | 0.833 (0.032) | 9.29×10^-9^ | 0.076 | 0.990 (0.050) | 0.842 | 5.452 | 1.031 (0.064) | 0.634 |
| rs10129827 | 14:67232573:A/G | 0.460 | 0.900 (0.028) | 1.57×10^-4^ | 0.065 | 1.253 (0.048) | 2.64×10^-6^ | 6.941 | 1.012 (0.063) | 0.846 |
| rs6573656 | 14:66711083:A/G | 0.432 | 0.899 (0.028) | 1.41×10^-4^ | 0.046 | 1.223 (0.051) | 8.15×10^-5^ | 5.749 | 1.037 (0.068) | 0.596 |
| rs9323497 | 14:67873128:T/C | 0.533 | 0.910 (0.028) | 6.47×10^-4^ | 0.065 | 1.298 (0.048) | 4.95×10^-8^ | 8.081 | 1.007 (0.063) | 0.911 |

## *Supplemental Table S6. Trans-ancestry meta-analysis results for females-only MDD.* Column headings are as described for Supplemental Table 5.

| SNP | Polymorphism | Discovery Phase | | | | | | | Replication Phase | |
| --- | --- | --- | --- | --- | --- | --- | --- | --- | --- | --- |
|  |  | CONVERGE | | | PGC | | | log_10_BF |  |  |
|  |  | Freq | OR (SE) | *P* | Freq | OR (SE) | *P* |  | OR (SE) | *P* |
| rs424175 | 1:163957374:T/C | 0.717 | 1.020 (0.031) | 0.515 | 0.902 | 0.762 (0.053) | 2.37×10^-7^ | 5.012 | 0.967 (0.068) | 0.620 |
| rs10912903 | 1:175161930:G/C | 0.418 | 0.865 (0.029) | 5.89×10^-7^ | 0.562 | 0.935 (0.029) | 0.020 | 5.426 | 0.980 (0.038) | 0.588 |
| rs12216613 | 7:5298791:A/T | 0.159 | 1.148 (0.036) | 1.31×10^-4^ | 0.376 | 1.143 (0.034) | 7.32×10^-5^ | 6.186 | 0.975 (0.041) | 0.531 |
| rs4515481 | 7:77850940:G/A | 0.213 | 0.887 (0.035) | 6.26×10^-4^ | 0.391 | 1.126 (0.030) | 5.59×10^-5^ | 5.186 | 0.984 (0.040) | 0.677 |
| rs145356089 | 10:69631362:T/C | 0.374 | 1.161 (0.028) | 9.75×10^-8^ | 0.015 | 0.844 (0.147) | 0.249 | 5.229 | 0.940 (0.156) | 0.693 |
| rs11245283 | 10:126236663:G/C | 0.318 | 0.846 (0.031) | 4.78×10^-8^ | 0.020 | 0.958 (0.141) | 0.759 | 5.735 | 1.021 (0.188) | 0.914 |
| rs10129827 | 14:67232573:A/G | 0.460 | 0.900 (0.028) | 1.57×10^-4^ | 0.065 | 1.260 (0.064) | 2.74×10^-4^ | 5.094 | 1.049 (0.081) | 0.560 |

##

## *Supplemental Table S7.* *Trans-ancestry meta-analysis results for recurrent MDD.* Column headings are as described for Supplemental Table 5.

| SNP | Polymorphism | Discovery Phase | | | | | | | Replication Phase | |
| --- | --- | --- | --- | --- | --- | --- | --- | --- | --- | --- |
|  |  | CONVERGE | | | PGC | | | log_10_BF |  |  |
|  |  | Freq | OR (SE) | *P* | Freq | OR (SE) | *P* |  | OR (SE) | *P* |
| rs4862792 | 4:188201350:G/T | 0.829 | 0.882 (0.039) | 1.13×10^-3^ | 0.873 | 0.852 (0.037) | 1.53×10^-5^ | 5.595 | 0.956 (0.054) | 0.399 |
| rs112182057 | 8:103518554:A/G | 0.023 | 0.780 (0.104) | 0.016 | 0.149 | 0.825 (0.040) | 1.32×10^-6^ | 5.768 | 0.965 (0.059) | 0.544 |
| rs145356089 | 10:69631362:T/C | 0.374 | 1.161 (0.028) | 9.75×10^-8^ | 0.015 | 0.905 (0.138) | 0.470 | 5.244 | 0.944 (0.148) | 0.697 |
| rs35936514 | 10:126244970:T/C | 0.301 | 0.833 (0.032) | 9.29×10^-9^ | 0.077 | 0.949 (0.056) | 0.351 | 5.981 | 1.013 (0.078) | 0.868 |
| rs28762177 | 14:67264567:T/G | 0.463 | 0.901 (0.028) | 1.69×10^-4^ | 0.065 | 1.216 (0.054) | 2.68×10^-4^ | 5.078 | 0.885 (0.076) | 0.108 |
| rs9323497 | 14:67873128:T/C | 0.533 | 0.910 (0.028) | 6.47×10^-4^ | 0.065 | 1.261 (0.054) | 1.46×10^-5^ | 5.620 | 0.875 (0.077) | 0.081 |
| rs66986914 | 18:2948741:C/A | 0.477 | 0.896 (0.028) | 6.72×10^-5^ | 0.132 | 1.136 (0.037) | 5.26×10^-4^ | 5.180 | 0.886 (0.055) | 0.027 |

## *Supplemental Table S8. Gene-set Enrichment Analysis with DEPICT.* For gene-sets with local FDR *q*-values less than 0.2, the *P*-value, *q*-value bin, and top 10 ranking genes (*Z*-score) are displayed.

| Diagnosis | GO category | Description | *P* | *q_FDR_* | Genes 1-10 (*Z-score*) |
| --- | --- | --- | --- | --- | --- |
| Lifetime MDD | GO:0021953 | central nervous system neuron differentiation | 5.01×10^-6^ | <0.05 | *DCC (4.4), NOVA1 (3.4), RUNX1T1 (3.3), ENSG00000254369 (3.0), CNTNAP2 (2.9), ELAVL2 (2.7), GPR98 (2.6), FGF19 (2.4), SERTAD4 (2.4), ENSG00000230268 (2.3)* |
|  | GO:0008088 | axon cargo transport | 6.50×10^-6^ | <0.05 | *ELAVL2 (2.9), ENSG00000234215 (2.9), CNTNAP2 (2.8), MAP1B (2.7), CRK (2.5), CTNNA3 (2.5), ENSG00000227868 (2.5), DIP2C (2.5), C12orf74 (2.5), DLG2 (2.5)* |
|  | GO:0010970 | microtubule-based transport | 3.86×10^-5^ | <0.20 | *DIP2C (3.3), CNTNAP2 (2.9), MAP1B (2.8), C6orf57 (2.3), FAM135A (2.2), NALCN (2.2), ELAVL2 (2.1), DLG2 (2.0, IQCH (2.0), STX8 (1.9)*) |
|  | MP:0001675 | abnormal ectoderm development | 1.05×10^-4^ | <0.20 | *CNTNAP2 (4.5), CXADR (2.7), FGF19 (2.7), CLVS2 (2.7), LUZP1 (2.5), GALNT13 (2.4), DIAPH3-AS1 (2.4), FGF3 (2.3), SIRT1 (2.2), FAM135A (2.2)* |
|  | MP:0000788 | abnormal cerebral cortex morphology | 1.35×10^-4^ | <0.20 | *OSTM1 (4.7), ELAVL2 (3.9), GRM3 (3.9), CNTNAP2 (3.6), GPR98 (3.0), ERBB4 (3.0), FGF19 (2.9), NRXN1 (2.8), SFRP1 (2.7), MAP1B (2.5)* |
|  | MP:0005458 | increased percent body fat | 1.42×10^-4^ | <0.20 | *VSTM2A (3.4), KCND3 (2.9), ZFPM2 (2.8), CLVS2 (2.6), NTN1 (2.6), KIAA0040 (2.5), GRM3 (2.4), FAM150B (2.4), LYG2 (2.3), DLG2 (2.2)* |
|  | ENSG00000173465 | SSSCA1 PPI subnetwork | 1.63×10^-4^ | <0.20 | *LHPP (3.4), IQCH (2.8), SCGBL (2.7), CCS (2.6), DEGS1 (2.4), ODF1 (2.4), WDR70 (2.3), RCE1 (2.2, ERBB4 (2.2), ARMC3 (2.1)* |
|  | MP:0000808 | abnormal hippocampus development | 1.72×10^-4^ | <0.20 | *DCC (4.0), C2orf15 (2.9), CLVS2 (2.9), CNTNAP2 (2.7), TMPRSS3 (2.7), ZFPM2 (2.6), FGF19 (2.4), KIT (2.3), CXADR (2.1), FAM150B (2.1)* |
|  | GO:0042430 | indole-containing compound metabolic process | 1.88×10^-4^ | <0.20 | *ENSG00000254235 (4.0), C12orf74 (3.3), SCGBL (3.0), ENSG00000226757 (2.8), C12orf54 (2.6), C10orf126 (2.6), CTNNA3 (2.5), ENSG00000258297 (2.5), DEGS1 (2.5), TAS1R2 (2.5)* |
|  | GO:0006586 | indolalkylamine metabolic process | 1.88×10^-4^ | <0.20 | *ENSG00000254235 (4.0), C12orf74 (3.3), SCGBL (3.0), ENSG00000226757 (2.8), C12orf54 (2.6), C10orf126 (2.6), CTNNA3 (2.5), ENSG00000258297 (2.5), DEGS1 (2.5), TAS1R2 (2.5)* |
|  | GO:0048638 | regulation of developmental growth | 2.29×10^-4^ | <0.20 | *FAM66C (3.8), CLLU1 (3.4), TMEM108 (3.3), CTNNA3 (3.2), PCDH7 (2.9), ZFPM2 (2.9), ENSG00000234817 (2.7), VSTM2A (2.7), ETV5 (2.6), C5orf42 (2.5)* |
|  | GO:0007628 | adult walking behavior | 2.42×10^-4^ | <0.20 | *FAM66C (4.3), C6orf208 (3.3), GALNT13 (3.0), KCNQ3 (2.9), NALCN (2.9), ENSG00000239332 (2.8), FSTL4 (2.8), GUCY1A2 (2.4), SLC25A26 (2.4), C21orf37 (2.4)* |
|  | GO:0023019 | signal transduction involved in regulation of gene expression | 2.61×10^-4^ | <0.20 | *FGF3 (4.7), DIAPH3-AS1 (3.4), FAM150B (3.3), LINC00290 (3.2), ENSG00000254652 (2.7), ENSG00000232013 (2.6), GRRP1 (2.5), FGF19 (2.4), LYG2 (2.3), TMPRSS3 (2.2)* |
|  | GO:0021761 | limbic system development | 3.24×10^-4^ | <0.20 | *OSTM1 (4.2), FSHR (3.6), DCC (3.2), DGKG (3.1), ENSG00000226757 (3.0), GUCY1A2 (2.8), CLVS2 (2.6), ENSG00000230417 (2.4), SLITRK1 (2.2), C6orf57 (2.2)* |
| Females-only MDD | ENSG00000174697 | LEP PPI subnetwork | 4.94×10^-5^ | <0.20 | *CLEC4A (3.6), ZC3H12D (3.0), KIAA0040 (2.6), EGFR (2.5), TMPRSS3 (2.5), C6 (2.5), C6orf120 (2.3), PON2 (2.2, ZNF793 (2.2), DNAJC12 (2.2)* |
|  | ENSG00000136488 | CSH1 PPI subnetwork | 6.53×10^-5^ | <0.20 | *CLEC4A (4.2), ZC3H12D (3.6), C6orf120 (2.8), TAB2 (2.5), GPR183 (2.5), ZNF793 (2.4), KIAA0040 (2.3), TMPRSS3 (2.2), EGFR (2.1), PDE4B (2.1)* |

# **References**

[1. Smith BH, Campbell H, Blackwood D, Connell J, Connor M, Deary IJ, *et al.* (2006): Generation Scotland: the Scottish Family Health Study; a new resource for researching genes and heritability. *BMC Med Genet*. 7: 74.](http://paperpile.com/b/Tlfggg/JhZh)

[2. Smith BH, Campbell A, Linksted P, Fitzpatrick B, Jackson C, Kerr SM, *et al.* (2013): Cohort Profile: Generation Scotland: Scottish Family Health Study (GS:SFHS). The study, its participants and their potential for genetic research on health and illness. *Int J Epidemiol*. 42: 689–700.](http://paperpile.com/b/Tlfggg/WzBM)

[3. Fernandez-Pujals AM, Adams MJ, Thomson P, McKechanie AG, Blackwood DH, Smith BH, *et al.* (2015): Epidemiology and Heritability of Major Depressive Disorder, Stratified by Age of Onset, Sex, and Illness Course in Generation Scotland: Scottish Family Health Study (GS:SFHS). *PLoS One*. 10: e0142197.](http://paperpile.com/b/Tlfggg/0TXr)

[4. Shi J, Potash JB, Knowles JA, Weissman MM, Coryell W, Scheftner WA, *et al.* (2011): Genome-wide association study of recurrent early-onset major depressive disorder. *Mol Psychiatry*. 16: 193–201.](http://paperpile.com/b/Tlfggg/evY0)

[5. Perlis RH, Iosifescu DV, Castro VM, Murphy SN, Gainer VS, Minnier J, *et al.* (2012): Using electronic medical records to enable large-scale studies in psychiatry: treatment resistant depression as a model. *Psychol Med*. 42: 41–50.](http://paperpile.com/b/Tlfggg/IGIj)

[6. Wray NR, Pergadia ML, Blackwood DH, Penninx BW, Gordon SD, Nyholt DR, *et al.* (2012): Genome-wide association study of major depressive disorder: new results, meta-analysis, and lessons learned. *Mol Psychiatry*. 17: 36–48.](http://paperpile.com/b/Tlfggg/KTcM)

[7. Major Depressive Disorder Working Group of the Psychiatric, Gwas Consortium, Ripke S, Wray NR, Lewis CM, Hamilton SP, Weissman MM, *et al.* (2013): A mega-analysis of genome-wide association studies for major depressive disorder. *Mol Psychiatry*. 18: 497–511.](http://paperpile.com/b/Tlfggg/yGaA)

[8. Breen G, Webb BT, Butler AW, van den Oord EJ, Tozzi F, Craddock N, *et al.* (2011): A genome-wide significant linkage for severe depression on chromosome 3: the depression network study. *Am J Psychiatry*. 168: 840–847.](http://paperpile.com/b/Tlfggg/uCbr)

[9. Cronin S, Berger S, Ding J, Schymick JC, Washecka N, Hernandez DG, *et al.* (2008): A genome-wide association study of sporadic ALS in a homogenous Irish population. *Hum Mol Genet*. 17: 768–774.](http://paperpile.com/b/Tlfggg/9R4d)

[10. Volzke H, Alte D, Schmidt CO, Radke D, Lorbeer R, Friedrich N, *et al.* (2011): Cohort profile: the study of health in Pomerania. *Int J Epidemiol*. 40: 294–307.](http://paperpile.com/b/Tlfggg/NfMT)

[11. Appel K, Schwahn C, Mahler J, Schulz A, Spitzer C, Fenske K, *et al.* (2011): Moderation of adult depression by a polymorphism in the FKBP5 gene and childhood physical abuse in the general population. *Neuropsychopharmacology*. 36: 1982–1991.](http://paperpile.com/b/Tlfggg/FaUn)

[12. Pruim RJ, Welch RP, Sanna S, Teslovich TM, Chines PS, Gliedt TP, *et al.* (2010): LocusZoom: regional visualization of genome-wide association scan results. *Bioinformatics*. 26: 2336–2337.](http://paperpile.com/b/Tlfggg/xcP5)
